# Free neighborhood choice boosts socially optimal outcomes in stag-hunt coordination problem

**DOI:** 10.1038/s41598-021-87019-y

**Published:** 2021-04-08

**Authors:** Arno Riedl, Ingrid M. T. Rohde, Martin Strobel

**Affiliations:** 1 Department of Microeconomics and Public Economics, Maastricht University, 6200 MD Maastricht, The Netherlands; 2 Faculty of Management, Open University of the Netherlands, 6401 DL Heerlen, The Netherlands; 3 Center for Economic Studies (CES), 81679 Munich, Germany; 4 IZA Institute of Labor Economics, 53113 Bonn, Germany; 5 Netspar, 5037 AB Tilburg, The Netherlands; 6 Institute for Applied Microeconomics, Bonn University, 53113 Bonn, Germany

**Keywords:** Psychology, Human behaviour

## Abstract

Situations where independent agents need to align their activities to achieve individually and socially beneficial outcomes are abundant, reaching from everyday situations like fixing a time for a meeting to global problems like climate change agreements. Often such situations can be described as stag-hunt games, where coordinating on the socially efficient outcome is individually optimal but also entails a risk of losing out. Previous work has shown that in fixed interaction neighborhoods agents’ behavior mostly converges to the collectively inefficient outcome. However, in the field, interaction neighborhoods often can be self-determined. Theoretical work investigating such circumstances is ambiguous in whether the efficient or inefficient outcome will prevail. We performed an experiment with human subjects exploring how free neighborhood choice affects coordination. In a fixed interaction treatment, a vast majority of subjects quickly coordinates on the inefficient outcome. In a treatment with neighborhood choice, the outcome is dramatically different: behavior quickly converges to the socially desirable outcome leading to welfare gains 2.5 times higher than in the environment without neighborhood choice. Participants playing efficiently exclude those playing inefficiently who in response change their behavior and are subsequently included again. Importantly, this mechanism is effective despite that only few exclusions actually occur.

## Introduction

The complexity of human societies requires both cooperation and coordination^1–4^. Most research has been devoted to the study of cooperation, the quest of overcoming social dilemmas by getting people to sacrifice for the greater good^5^. However, many challenges of human interaction have the structure of a coordination problem, that is, the problem of aligning activities of independent agents to achieve beneficial outcomes for all^1,6^. Examples of coordination problems are abundant, reaching from daily-life problems like deciding which way to move when two people meet on a path, questions of technology adoption, speculative exchange rate attacks and inter-sectoral coordination in macroeconomics, aligning activities of different layers in multilevel governance, the efficient integration of activities of different parts of organizations, to climate change agreements^7–14^. It also has been shown that social dilemmas are effectively transformed into coordination problems when players have altruistic or other-regarding preferences^15^ and recent research in psychology reports that people perceive most of their social interactions as one resembling the stag-hunt game, a prototypical coordination problem^16^. In philosophy it has been suggested that the social contract is best discussed using the stag-hunt game, an idea dating back to Jean-Jacques Rousseau^17^. Despite its recognized importance, relatively little is known about how to make people coordinate efficiently.

In stag-hunt coordination games, the attempt to coordinate on an individually and collectively optimal solution exposes players to the risk of a loss. Individual agents might therefore opt for a collectively inefficient but safer outcome^18^. Indeed, theoretical work^19–22^ as well as experimental investigations^23–25^ have shown that, in the long run, overwhelmingly the safe but inefficient outcome emerges, except when groups are very small^23,26^. This attraction to the inefficient outcome is socially undesirable and the question arises whether there is a way to help individuals to robustly coordinate on the individually and collectively optimal outcome. Here we provide evidence that giving individuals the freedom to choose their interaction neighborhood achieves this, thereby boosting individual and social welfare.

An important restriction of the mentioned studies is that they assume that individuals interact in fixed neighborhoods and thus cannot choose with whom to coordinate. However, in the field in most social interactions agents can choose (at least to some extent) their interaction neighborhood and it seems natural to ask if that could help to overcome inefficient outcomes. Theoretical work that has examined the effect of more fluid interaction structures has arrived at contradicting results. Some research suggests that the possibility of neighborhood choice increases the chances for behavior to converge to the efficient outcome^27,28^ while other work predicts that the safe but inefficient outcome will prevail^21,22,29,30^. It is thus an empirical question whether free neighborhood choice is a mechanism that can implement socially efficient outcomes in coordination problems.

Recently, for social dilemmas (Prisoners’ Dilemma and Public Goods Game) experimental evidence indicates that more fluid interaction structures can but need not be beneficial. Although suggestive, the evidence from social dilemma experiments is not immediately informative for coordination problems. First, although problems of cooperation and coordination appear similar, the incentive structures differ substantially and it is unknown if results from social dilemma problems carry over to coordination problems. Second, the reported effects of fluid interaction structures in social dilemma games are not uniformly and strongly positive. Most studies report mildly positive effects of dynamic interaction structures on cooperation that are, however, far off the fully efficient outcome^31–35^ or—with one exception—not maintained in the long run^36,37^. Thus, direct evidence is needed on whether free choice of the interaction neighborhood helps individuals to coordinate fully and robustly on an efficient outcome in coordination problems. Such evidence is largely missing, though see^38,39^.

We examined the effect of neighborhood choice on the efficiency of coordination by letting individuals play stag-hunt games in groups of six. In a baseline treatment, called Imposed, all six individuals were forced to play with each other, implying that each subject played five pair-wise stag-hunt games. In line with proposed theoretical models^29,30^, each individual could choose to play either efficiently (blue choice in the experiment) or safe (green choice) with all of the other five individuals in the group. The payoffs in each of the pair-wise stag-hunt games were as follows. In case both individuals in a pair chose the efficient action each earned 95 points, in case both chose the safe option each earned 75 points, and in case one chose the efficient and one the safe option the former earned 5 points whereas the latter earned 90 points. The total payoff of a player was the sum of payoffs in all pair-wise interactions. Clearly, everyone playing the efficient action is a strict Nash equlibrium, which is individually and collectively optimal. Everyone playing the safe option is also a Nash equilibrium which is however inefficient. Importantly, all individuals had to choose their action simultaneously which introduces strategic uncertainty. Specifically, choosing the efficient action was a best response, only when a (risk neutral) individual expected that all other five individuals also choose the efficient action. We chose this strong incentives for safe play deliberately to make it hard to achieve the efficient outcome.

To test if freedom to choose the interaction neighborhood increases the efficiency of coordination we conducted a treatment called Free. This treatment was exactly the same as Imposed, except that with the action choice in the stag-hunt games, all individuals in a group had to simultaneously propose with whom in their group they wanted to interact. Each individual could propose to interact with any of the other individuals in their group and only if both individuals in a pair proposed to interact this pair actually played a pair-wise game. Note that when all individuals in a group proposed to interact with everyone else in the group then each individual played five pair-wise stag-hunt games and the interaction neighborhood was exactly the same as in Imposed. Importantly, irrespective of the outcome in the stag-hunt game, not interacting in a game was costly because in that case both individuals in the respective pair earned zero points. This is an important difference from reported social dilemma games with dynamic interaction structures where not interacting is more beneficial than some outcomes in the social dilemma (see “Discussion”). To allow for learning and to investigate the dynamics of stag-hunt game actions and interaction neighborhoods, in both treatments each group played the games repeatedly for 30 rounds.

**Figure 1:**
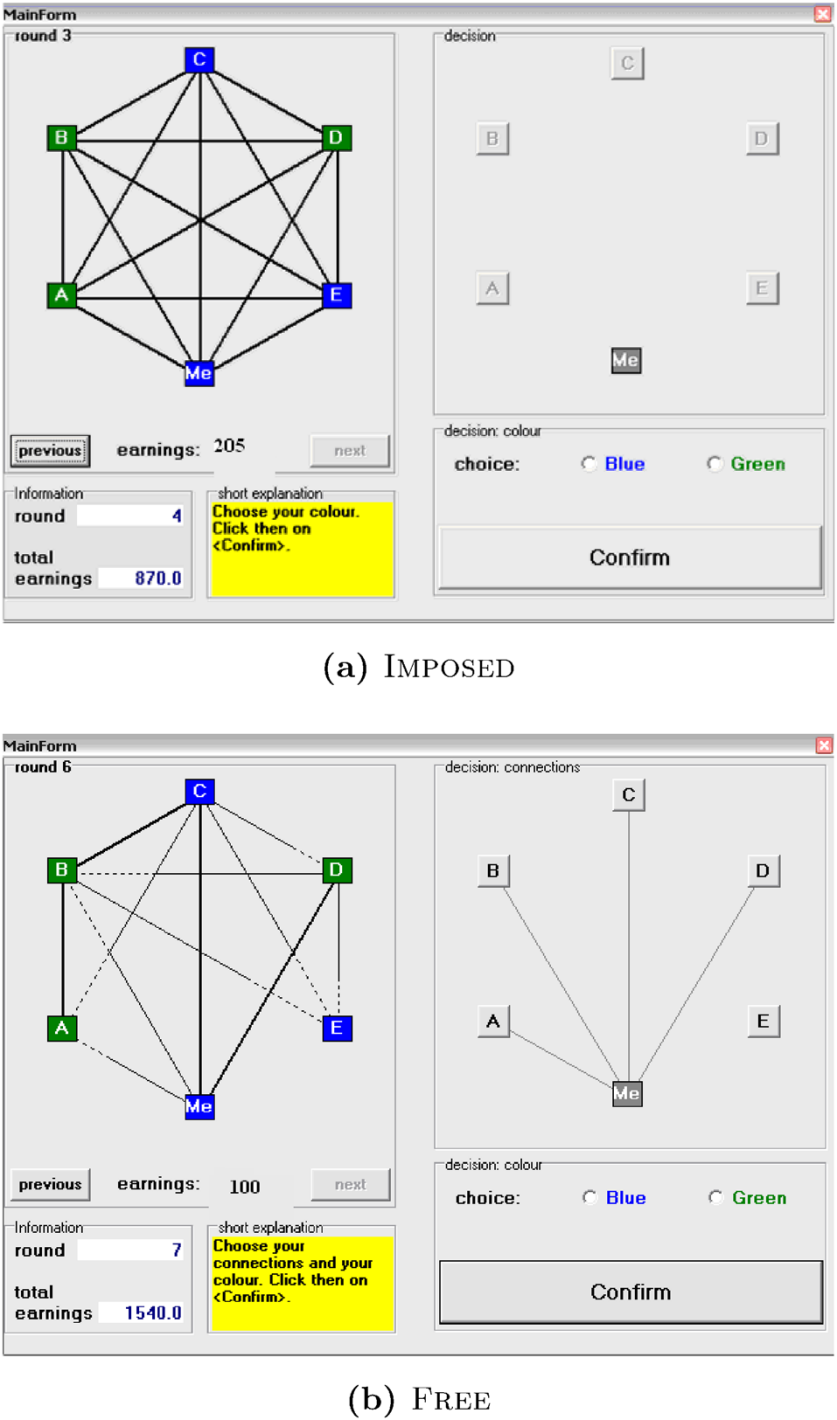
Information and decision screens in Imposed and Free. In both treatments the left panel provided information of behavior in previous rounds, with colors (blue, green) indicating the chosen action in the stag-hunt games, as well as earnings in each round and cumulative earnings. Action decisions were made on the right panel by clicking on button Blue or Green. (**a**) In Imposed all group members were forced to interact with each other which is indicated by the thick full lines between all pairs of group members. (**b**) In Free, next to action choices also proposals to interact were made on the right panel by clicking on the letter buttons. Lines indicated interaction proposals. On the left panel thick full lines indicated pair-wise interactions that took place and broken lines non-reciprocated interaction proposals. In the example, only ‘C’ and ‘D’ are in the interaction neighborhood of ‘Me’.

The experiment was implemented in six sessions, three sessions of Imposed and three sessions of Free. In total, $n=108$ subjects (median age 22; $43\%$ female), equally and randomly distributed across the two treatments, participated in the experiment. In each session, 18 subjects were divided at random into groups of six. The groups stayed together for all 30 rounds of the stag-hunt games. Participants were seated at sight-shielded computer cubicles where they read the experiment instructions (see “Methods” and Supplementary Information (SI)). In treatment Imposed, at the beginning of each round each participant in a group had to choose either the blue (efficient) or green (safe) action. At the end of each round, each individual in a group was anonymously informed of the choices of all other group members (Fig. 1a). In treatment Free, each group member had to propose their interaction neighborhood next to the action choice in the stag-hunt game, in each round. Only if an interaction proposal was reciprocated group members were in each others interaction neighborhood. At the end of each round, each group member was informed about the established interaction neighborhoods and the actions chosen by all group members (Fig. 1b).

Our main research question is whether interaction in an imposed neighborhood will produce socially undesirable outcomes, i.e., inefficient coordination or even miscoordination, whereas the freedom to choose ones interaction neighborhood will facilitate efficient coordination. To examine this we compare if coordination outcomes differ between the Imposed neighborhood treatment and the Free neighborhood choice treatment. We will first look at the aggregate picture and then zoom into the dynamics of behavior. In addition, we will explore the behavioral mechanism underlying the results.

In our study each group forms a strictly independent observation. Unless otherwise indicated, the reported *p*-values are based on appropriate regression models (logit, tobit) with robust standard errors corrected for data dependence within groups. In the SI we also report statistics based on more conservative non-parametric tests with aggregate group measures as the unit of observation. Notice that the correction and aggregation at the group level leaves us with $n = 9$ strictly independent observations per treatment so that the detection of a statistically significant effect is indicative of a large treatment effect. Individual group outcomes are reported in the SI.

## Results

Our main measures of coordination success and failure are the frequency of (1) socially desirable (efficient) coordinated outcomes, (2) socially undesirable (inefficient) coordinated outcomes, and (3) socially undesirable miscoordination. Coordination in a pair of players (dyad) is called efficient when both subjects choose the efficient action, inefficient when both subjects choose the inefficient action, and there is miscoordination when they choose different actions. For Free we also look at the frequency of no-play, that is, when at least one subject in a dyad does not propose to interact with the other subject. Note that no-play implies zero earnings for both subjects involved, whereas interacting leads to positive earnings for both irrespective of the actions chosen in the stag-hunt game. Thus, from an individual perspective it is always optimal to propose to interact.

### The (in)efficiency of coordination outomes

**Figure 2:**
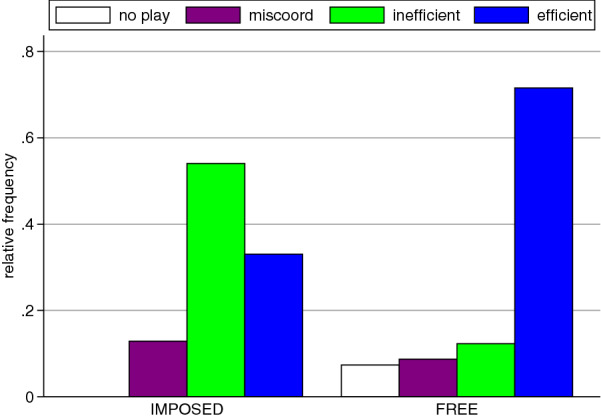
Frequency of the different outcomes in imposed neighborhoods (Imposed) and free neighborhood choice (Free) aggregated over all rounds. Inefficient coordination (green) is much more frequent in Imposed than in Free, whereas efficient coordination (blue) is much more frequent in Free than in Imposed. Miscoordination (purple) is slightly more frequent in Imposed than in Free. No-play (white) can only occur in Free and is infrequent.

Aggregated over all rounds, we find that when subjects interact in an Imposed neighborhood, inefficient coordination is most frequent (54%), efficient outcomes are relatively infrequent (33%), and miscoordination occurs 13% of the time (Fig. 2 Imposed). Thus, in two-thirds of all cases outcomes are socially undesirable. These somber result is consistent with previous evidence from coordination games in imposed neighborhoods^25,40^.

The picture changes dramatically with Free neighborhood choice. In this treatment, inefficient coordination occurs in only 12% of all cases, and miscoordination and no-play are also infrequent (9% and 7%, respectively). In contrast, socially desirable efficient coordination is the prevalent outcome, observed in 72% of all cases (Fig. 2 Free). Comparing Imposed and Free, shows that the differences in frequencies of both efficient and inefficient coordination are statistically significant (efficient coordination: $p=0.039$, logit; inefficient coordination: $p=0.025$, logit). The frequencies of miscoordination do not differ significantly ($p=0.203$, logit). Thus, free neighborhood choice mitigates inefficient actions in the stag-hunt games and boosts efficient outcomes.

These differences between Imposed and Free do not emerge immediately. In the first round, inefficient coordination is less prevalent in Free than in Imposed (10% vs. 25%, $p=0.064$, logit) but the frequency of efficient outcomes is not significantly different (Imposed 33%, Free 41%, $p=0.612$, logit). In the beginning, there is also substantial miscoordination which is similar in both treatments (Imposed 42%, Free 35%; $p = 0.431$; logit). This suggests that the clear overall differences in outcomes are mostly due to treatment specific dynamics. We note that in round 1 individual data are independent and we therefore do not need to correct for dependency within interacting groups. Thus, the insignificant results are unlikely due to missing statistical power.

**Figure 3:**
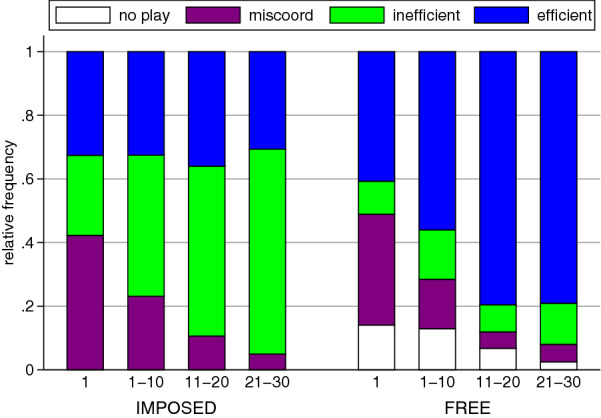
Frequency of the different outcomes in Imposed and Free in round 1 and aggregated over rounds 1–10, 11–20, and 21–30. In round 1 there is little difference in outcomes between treatments. Over rounds inefficient coordination (green) becomes more frequent in Imposed whereas efficient coordination (blue) becomes more frequent in Free. Miscoordination (purple) decreases in both treatments. No-play (white), only possible in Free, disappears over time.

Figure 3 depicts the frequencies of the different outcomes for the first round and for blocks of 10 rounds (i.e., rounds 1–10, rounds 11–20, and rounds 21–30). It shows that in both treatments the frequency of miscoordination decreases over rounds (both treatments: $p<0.001$; $n=9$, Jonkheere-Terpstra (JT) tests for trends^41^). However, the direction of the dynamics sharply differs between Imposed and Free. In the former, the frequency of socially undesirable inefficient outcomes increases, whereas in the latter it is the frequency of the socially desirable efficient outcomes that increases ($p=0.002$ and $p<0.001$; JT tests). Over time, the frequency of efficient outcomes does not change much in Imposed and, in Free the frequency of inefficient outcomes is stable at a low level. Notice that in Free, where at the beginning some interactions do not take place (14% in round 1), subjects quickly learn to avoid this outcome. Over rounds, the frequency of no-play decreases significantly ($p<0.001$; JT test) and vanishes almost completely towards the end of the 30 rounds.

These different dynamics in outcomes lead to increasing differences between Imposed and Free. In rounds 1–10 the frequencies of the different outcomes do not yet strongly differ between treatments (efficient: $p=0.204$, inefficient: $p=0.061$, miscoordination: $p=0.210$; logit). However, in later rounds statistically significant differences emerge for both, efficient and inefficient outcomes (efficient: $p=0.031$ (rds. 11–20), $p=0.020$ (rds. 21–30); inefficient: $p=0.036$ (rds. 11–20), $p=0.032$ (rds. 21–30); miscoordination: $p=0.225$ (rds. 11–20), $p=0.879$ (rds. 21–30); logit). Note that in both treatments the frequency of miscoordination clearly decreases with rounds. Thus, the stark overall differences in efficient and inefficient coordination between Imposed and Free are created by miscoordination being turned into coordinated inefficient outcomes in the former but coordinated efficient outcomes in the latter.

### Inclusion and exclusion of interaction partners

What is the mechanism behind this divergence? Treatment Free differs from Imposed only in the fact that in the former individuals can choose their interaction neighborhood. The answer to the question must thus be found there. Therefore, we now turn to a detailed analysis of the dynamics of dyadic relationships in the Free neighborhood choice treatment. We compare dyadic relations in round $t-1$ and the subsequent round *t* and explore two measures: *inclusion* and *exclusion* of other group members. We say that an individual *includes* another individual in round *t* when s/he proposes to interact with this individual in *t* while not having interacted with him or her in $t-1$. Similarly, we say that an individual *excludes* another individual in round *t* if s/he does not propose to interact with this individual in *t* while having interacted with him or her in $t-1$.

**Figure 4:**
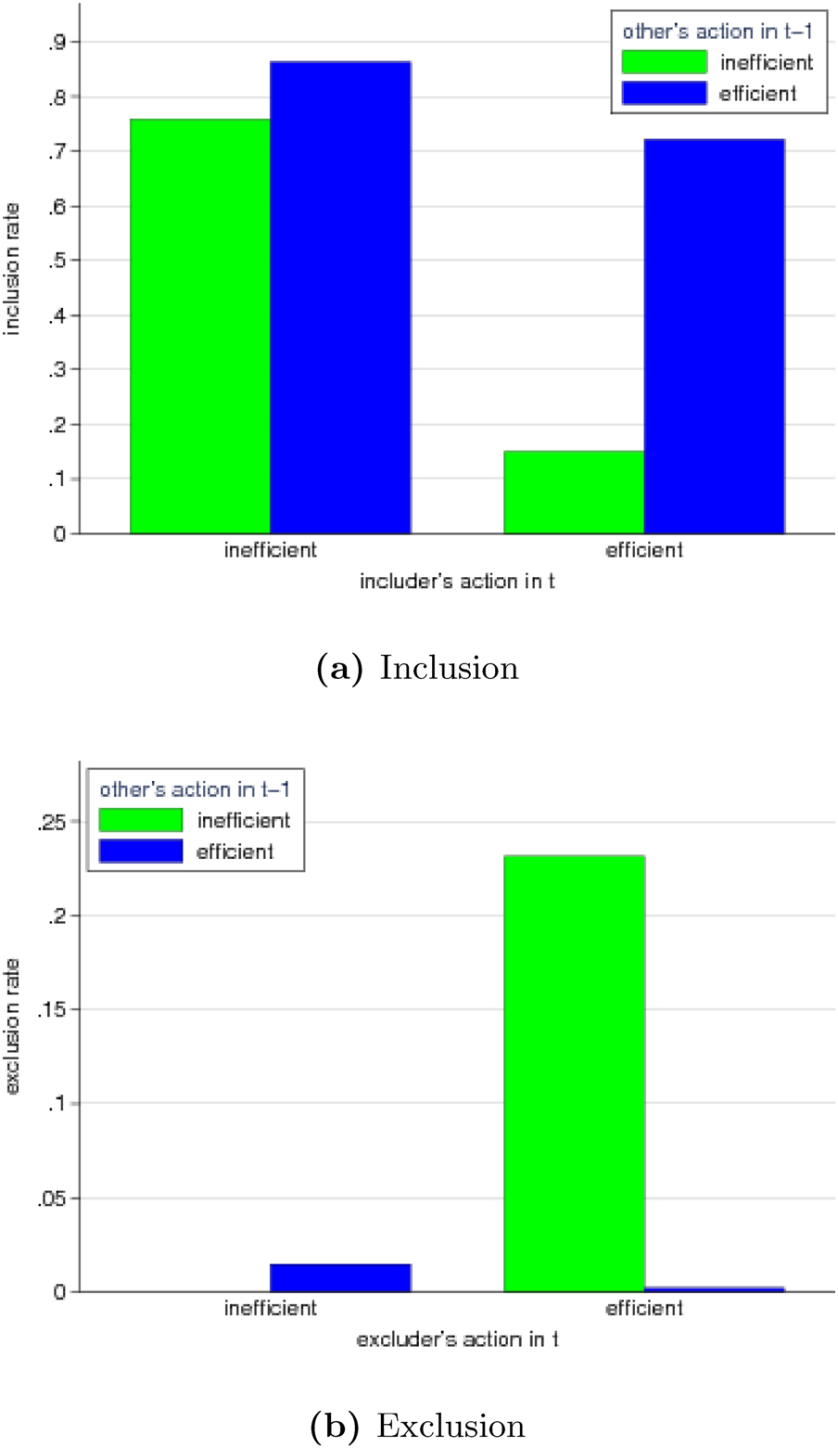
Inclusion and exclusion after efficient and inefficient actions in the coordination game. (**a**) Inclusion: Subjects are more likely to be included in round *t* when they chose the efficient action (blue) than when they chose the inefficient action (green) in round $t-1$. This discrimination is more pronounced when the potential ‘includer’ chooses the efficient action (right pair of bars) than when s/he chooses the inefficient action (left pair of bars). (**b**) Exclusion: Subjects playing the efficient action in round *t* (right pair of bars) frequently exclude other subjects who chose the inefficient action in the previous round (green) but not if they chose the efficient action (blue). Subjects playing the inefficient action in round *t* almost never exclude other subjects, irrespective of those subjects’ previous action choices (left pair of bars).

Subjects show a general tendency to include those they did not interact with before. The overall inclusion rate amounts to 63%. However, inclusion is not unconditional but depends on a subject’s own action in the coordination game as well as on the previous action of the (potential) interaction partner (Fig. 4a). A subject choosing the efficient action in period *t* includes another subject that chose the efficient action in $t-1$ in 72% of the cases. This percentage strongly drops to only 15% when the prospective interaction partner chose the inefficient action in $t-1$. Hence, subjects intending to play efficiently clearly discriminate between prospective interaction partners by taking into account those previous actions ($p<0.001$, logit). For subjects who themselves choose the inefficient action in *t* we also see such discriminatory behavior, which is much less pronounced however. They include subjects who previously chose the efficient action in 86% of the cases and those who previously chose the inefficient action in 76% of the cases ($p=0.012$, logit).

When it comes to exclusion, discrimination between prospective interaction partners strongly depends on whether subjects themselves play the inefficient or the efficient action (Fig. 4b). Subjects who choose the inefficient action in a round are virtually never excluding other subjects, irrespective of those subjects’ previous action in the coordination game (0% and 1.5% in case the prospective interaction partner chose the inefficient and efficient action, respectively). In contrast, subjects playing the efficient action, exclude other subjects who chose the inefficient action in the previous round in 23% of the cases, whereas exclusion of subjects who chose the efficient action in the previous round is virtually absent ($<0.2\%$). This difference in exclusion rates is statistically significant ($p<0.001$, logit). In other words, subjects choosing the efficient action are willing to forgo the gains from interacting and frequently exclude inefficient subjects whereas those who play inefficient themselves are unwilling to do so.

### The effect of inclusion and exclusion on coordination outcomes

The presented evidence suggests that inclusion and exclusion of (prospective) interaction partners is the mechanism behind the high rate of efficient outcomes in Free. However, the question remains whether inclusion and exclusion in a round indeed lead to more efficient actions in future rounds. To analyze this we extend our analysis of the dyadic relations to round $t+1$ following inclusion or exclusion. Specifically, we explore whether subjects who are included or excluded in round *t* change their behavior in round $t+1$ relative to round $t-1$, the round that may have triggered inclusion or exclusion. Before presenting these results it is interesting to note that overall subjects exhibit quite some inertia in their behavior. The rate of efficient actions in $t+1$ is 96% after an efficient action in $t-1$ and only 21% after an inefficient action in $t-1$ ($p<0.001$, logit).

**Figure 5:**
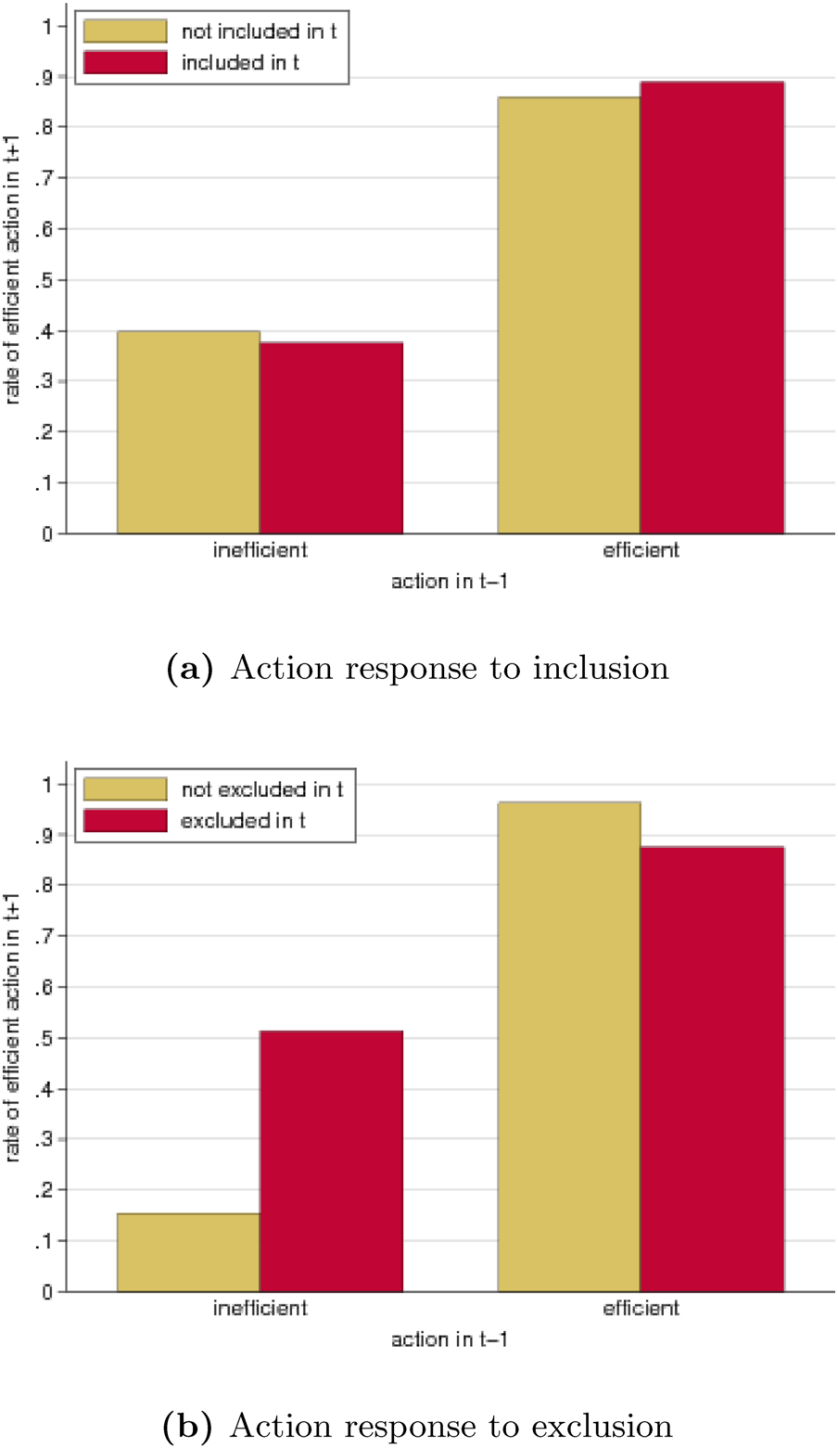
Efficient and inefficient actions after inclusion and exclusion. Overall subjects are more likely to choose the efficient action when they had chosen this action before (compare left and right pairs of graphs). (**a**) Action response to inclusion: The frequency of efficient actions is almost identical after no inclusion (yellow bars) and after inclusion (red bars), irrespective of whether an inefficient or efficient action was chosen in the past. (**b**) Action response to exclusion: Subjects excluded after an inefficient action are much more likely to switch to the efficient action (left red bar) than those not excluded (left yellow bar). Subjects excluded after an efficient action are slightly less likely to choose the efficient action (right red bar) than those not excluded (right yellow bar).

Inclusion barely affects subjects’ likelihood to choose the efficient action (Fig. 5a). After an inefficient action in $t-1$, the rate of efficient actions in $t+1$ amounts to 38% in response to inclusion but is 40% when a subject was not included ($p=0.928$, logit). After an efficient action in $t-1$, the rate of efficient actions in $t+1$ is 89% in response to inclusion and amounts to 86% when a subject was not included ($p=0.345$, logit).

The action response to exclusion is very different (Fig. 5b). After an inefficient action in $t-1$ the rate of efficient actions in $t+1$ after exclusion in *t* anounts to 51%, while this rate is only 16% when no exclusion took place ($p<0.001$, logit). Subjects who had chosen the efficient action already in $t-1$ are barely excluded in *t* (cf. Fig. 4b). In the rare cases where it does happen it has a small adverse effect on the frequency of efficient actions: after exclusion the rate of efficient actions in $t+1$ amounts to 88%, whereas after no exclusion it is 96% ($p=0.031$, logit).

Thus, there is clear evidence that subjects use the freedom to choose their interaction neighborhood to include subjects previously playing the socially desirable action and to exclude those who opted for the socially undesirable action. The latter respond to exclusion by switching to the efficient action. Together with the high likelihood to stick with the efficient action once it has been chosen this response to exclusion boosts efficient coordination and stabilizes it at a high level. Hence, the driving force behind the high level of efficient coordination in Free is exclusion of subjects who have chosen the inefficient action in earlier rounds.

Importantly, exclusion implies that not all coordination games that could have been played are actually played. These not played games return zero earnings, whereas any played coordination game leads to positive earnings, even if the outcome is miscoordindation. Thus, exclusion is individually and socially costly and the question arises whether free neighborhood choice not only boosts efficient coordination but also increases welfare.

### Welfare effects

To explore the welfare effects of free neighborhood choice we compare earnings on the dyad level in Free and Imposed over time. To this end, we define the welfare gain relative to the earnings in the inefficient equilibrium and normalize it to the difference between earnings in the efficient and inefficient equilibrium. Formally, let *X* be the sum of actual earnings in a dyad, $\Pi _{e}$ the sum of earnings in a dyad when coordinating on the efficient equilibrium, and $\Pi _{ie}$ the sum of earnings in a dyad when coordinating on the inefficient equilibrium. The normalized welfare gain is then defined as $(X-\Pi _{ie})/(\Pi _{e}-\Pi _{ie})$, which can be negative and runs from $-3.75$ when a dyad does not interact and both subjects involved earn zero (the worst possible outcome in Free) to $+1$ when both subjects in a dyad choose the efficient action. The worst possible outcome in Imposed is miscoordination and represented by a welfare loss of 1.375 (i.e., a welfare ‘gain’ of $-1.375$). In terms of welfare comparison, this puts Free in a disadvantaged position as the best outcome in Free and Imposed are identical while the worst outcome is much worse in Free than in Imposed.

**Figure 6:**
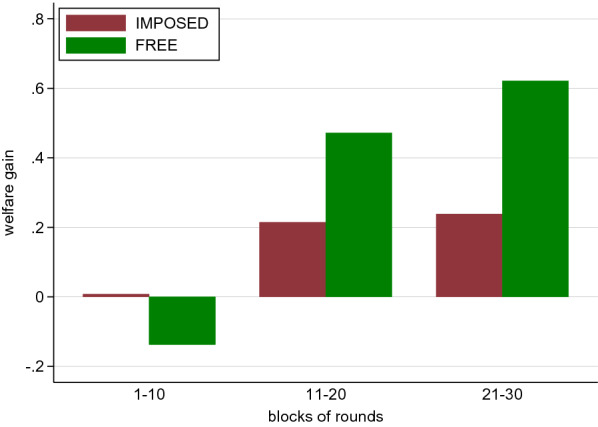
Welfare gains.

The dynamics of welfare gains over time differ quite strongly between treatments (Fig. 6). In the beginning (rounds 1–10), the welfare gain is with 0.01 virtually zero in Imposed, whereas there is a slight welfare loss in Free ($-0.14$). These early-rounds welfare gains are indistinguishable from zero, the level of the inefficient equilibrium ($p \ge 0.243$, tobit). The welfare loss in Free suggests that exclusion tends to be socially costly, in the early rounds. The difference between Imposed and Free is not significant though ($p=0.819$, tobit). Over time welfare gains grow larger in both treatments but they grow faster in Free than in Imposed. For intermediate rounds (11–20), the welfare gain in Free amounts to 0.47, whereas in Imposed it is only 0.21 and thus more than 50% smaller. In Imposed the welfare gain is not significantly different from the inefficient equilibrium level of zero ($p=0.178$, tobit), whereas it is significantly larger in Free ($p=0.013$, tobit). The difference between treatments is significant at $p=0.075$ (tobit). This difference in treatments is further reinforced in the last third of rounds (rounds 21–30). There the welfare gain in Free grows up to 0.62 whereas the development in Imposed stagnates at 0.24 and this difference is significant ($p=0.020$, tobit). Furthermore, in Imposed the welfare gain still does not differ from the inefficient equilibrium level ($p=0.165$, tobit) whereas in Free welfare is significantly larger than the inefficient equilibrium level ($p<0.004$, tobit). Thus, the freedom to choose the interaction neighborhood leads to welfare gains, especially in the longer run.

## Discussion

We started out by asking if the freedom to choose the interaction neighborhood can overcome the frequently observed inefficient coordination in stag-hunt coordination games. The answer to this question is clearly affirmative. With no neighborhood choice actions in the coordination game converge towards the inefficient equilibrium, whereas with neighborhood choice subjects overwhelmingly choose the efficient action. Noteworthily, the effect is not immediate but kicks in over time. In the beginning the frequencies of efficient actions and inefficient actions are similar with and without neighborhood choice. What makes the difference is that the initially relatively frequent miscoordination outcomes develop into inefficient coordination when there is no neighborhood choice but into efficient coordination when neighborhood choice is possible. Importantly, these different dynamics in behavior also result in significant and substantial differences in welfare. While in the beginning welfare gains are similar in both environments, neighborhood choice quickly leads to increasing welfare gains. In the last phase of the experiment these welfare gains are 2.5 times larger with than without neighborhood choice.

We have identified exclusion as the driving force behind the efficient coordination in the environment with neighborhood choice. Subjects who themselves choose the efficient action in the coordination game, discriminate between other subjects on the basis of those subjects’ earlier action choices: they include those who chose the efficient action and exclude those who chose the inefficient action. Interestingly, only exclusion induces behavioral change, while inclusion bears little effect. Excluded subjects who previously chose the inefficient action subsequently switch to playing the efficient action. Together with substantial behavioral inertia, that is, subjects tend to stick to the efficent action once they have chosen it, this triggers the high frequency of efficient coordination.

Importantly, the strong effect of neighborhood choice on efficient coordination is not driven by massive exclusion. When neighborhood choice was possible, a staggering 93% of all possible coordination games is actually played, meaning that in almost all instances the endogenously created interaction structure coincides with the one in the environment where subjects were forced to interact with each other. Thus, the dramatic difference in efficient coordination between the two investigated environments is driven by only 7% of all interaction choices. Most of these occur in the early rounds (rounds 1–10: 12.9%, rounds 11–20: 6.7%, rounds 21–30: 2.5%). This indicates that early and even very infrequent actual exclusion is sufficient to boost efficient coordination.

Our study is related to work exploring how exogenously changing group sizes can facilitate efficient coordination^42^. There it has been shown that when groups start at size 2, slowly growing the group size can sustain efficient coordination also in larger groups. In our experiment groups were of size 6 and efficient coordination quickly broke down in the imposed interaction neighborhood. When there was free neighborhood choice subjects could in principle first choose small interaction neighborhoods and than slowly grow them to full size while maintaining efficient coordination. However, this is not what we observe. When there is free neighborhood choice, already in the first round 86% of all possible interactions actually take place (cf. Fig 3). That is, on average in the first round the size of the interaction neighborhood was already above 4 (of a maximum of 5). In later rounds it never fell below 3.9 (round 4) and remained close to 5 in all other rounds (rounds 1–10: 4.4, rounds 11–20: 4.7, rounds 21–30: 4.9). This means that in most rounds all members of a group interacted with everyone in the group, just like when no neighborhood choice was possible. Nevertheless, the flexibility of excluding and including group members one-by-one appears to be important for the effectiveness of free neighborhood choice. Therefore, future research could investigate if restricting this flexibility affects the efficiency of coordination.

Another aspect that may warrant future research is the value of the payoff one receives when not being in a neighborhood of another player. That is, when not playing the stag-hunt game because of being excluded by others or because oneself severed a neighborhood link. In our set-up, subjects who do not play the stag-hunt game receive a payoff of zero, which is lower than any payoff from participating in it. Specifically, the payoff one receives when playing the efficient action (Blue) while the other player plays the inefficient action (Green) is with a value of 5 larger than the outside payoff 0. Thus, there is an incentive to (try to) reenter the stag-hunt game, irrespective of the action of the other subjects. One may wonder whether our main result—that neighborhood choice boosts efficient outcomes—is robust to a change in the relation between these two payoffs. In particular, one may speculate, whether subjects may avoid to rejoin the stag-hunt game, when the payoff from playing the efficient action, while the other is (expected of) playing the inefficient action, is negative, or more generally below the payoff of not participating in the stag-hunt game. If that would be the case, it could undermine the observed positive effect of neighborhood choice.

There are at least two reasons why we consider it unlikely that the discussed change in payoffs would significantly weaken the positive effect of neighborhood choice. First, to not join the neighborhood of another subject, the subject in question needs to expect that the other subject does not play the efficient action. Looking at our data this is very unlikely to happen, however. In an overwhelming majority of cases ($82\%$) it is those playing the efficient action who exclude players who previously chose the inefficient action. Due to the observed substantial inertia in behavior, the former are highly likely ($87\%$) to stick to the efficient action when offering to include formerly excluded players again. Thus, a subject who decides to reenter the stag-hunt game almost surely meets a player choosing the efficient action, which generates a substantial payoff gain over staying out. Therefore, a player who chooses the efficient action would not have an incentive to reenter the stag-hunt game, only if the payoffs from meeting a player choosing the inefficient action would be unrealistically low. Second, in our set-up the earnings when staying out are below all payoffs when playing the stag-hunt game. That is, the act of exclusion is always costly, which makes it relatively unattractive. Consistent with this, observed actual exclusion rates are with 7% very low—but nevertheless very effective in triggering the choice of the efficient action and thus efficiency enhancing. When the payoff for a player choosing the efficient action, who meets a player choosing the inefficient action, would be negative, it would be actually beneficial for the former to exclude the latter. Therefore, one may expect a higher frequency of (early round) exclusions of players choosing the inefficient action, which will likely trigger them to switch to the efficient action even more than in the original set-up.

Yet, there are certainly limits to the effectiveness of neighborhood choice, similar to the effectiveness of punishment in cooperation games^43^. For instance, if the payoff of playing efficiently, while the opponent plays inefficiently is extremely low (e.g., far below $-95$ in our set-up), then players may not want to take the risk at all and stay out of the stag-hunt game. Alternatively, if the act of neighborhood choice is very costly (i.e., if staying out leads to payoffs far below all payoffs achievable in the stag-hunt game), neighborhood choice may not happen at all. Thus, exploring the limits of the effectiveness of neighborhood choice appears as a worthwhile avenue of future research.

Our study is also related to work investigating the role of dynamic interaction structures in social dilemma problems. There it has been shown that dynamic interactions can increase the incidence of cooperation. Importantly, in most of these studies the observed increase in cooperation is relatively mild or not long lasting, whereas we see a strong and robust increase in efficient coordination. Moreover, next to the fact that a coordination problem is not directly comparable to a cooperation problem, the results of these social dilemma studies cannot be straightforwardly extended to our study for several reasons. First, many of these studies do not allow for free neighborhood choice as we do but enforce dynamic interaction structures exogenously^4,31,32^. Second, most of these studies implement, implicitly or explicitly, an infinitely repeated game by either not telling subjects the number of total rounds or implementing a random ending^4,31,32,34,35,37^, which does not allow to investigate whether dynamic interaction structures also affect behavior when subjects know the end of the game. In fact, the social dilemma study closest to our research that implements a known horizon, finds that in most cases cooperation breaks down completely already well before the game ends^36^. In contrast, we find that even with a known horizon efficient coordination can be upheld until the very end. Third, in all social dilemma studies not playing the game was never the worst outcome and in most studies it was (weakly) better than mutual defection. This feature made it relatively cheap or even beneficial to exclude defectors. In contrast, in our study exclusion was costly irrespective of the outcome in the stag-hunt game making exclusion a very unattractive option from an earnings perspective.

Despite these differences the reported social dilemma studies are informative for potential future research on coordination problems. For instance, it has been shown that the effectiveness of fluid interaction structures in promoting cooperation sometimes hinges on details: when interaction neighborhoods can be updated only infrequently, cooperation tends to break down when players where offered random opportunities to make or break interaction links^32^ but tends to stay high when players can choose themselves with whom they want to interact^36^. For large groups there is also evidence that cooperation is best achieved at intermediate levels of changes of interaction partners^33^. How the frequency of interaction partner update affects behavior in coordination games is unexplored territory. One may conjecture that in coordination problems the achieved efficiency is less sensitive to changes in the frequency of neighborhood choice because coordination on the efficient outcome is a Nash equilibrium. However, we also know that human players are very sensitive to potential losses and may thus choose the safe inefficient action when the possibility to reduce strategic uncertainty via neighborhood choice is available only infrequently.

Other work on social dilemma problems has pointed at the potential importance of the information players receive and the initial interaction structure they start with^34,37^. In our experiment, subjects had access to past action choices of all other group members as well as the established and proposed interaction neighborhoods. As identifying those who chose inefficiently in the past could be crucial for the effectiveness of exclusion in boosting efficient coordination varying this information could be an important robustness check for our main result. Further, although it is well known that efficient coordination breaks down already for relatively small groups of size four^23^ it remains to be seen if the positive effect of free neighborhood choice is robust to larger groups of hundreds or thousands of potential interaction partners^4,37^.

We investigate a strategic environment with multiple players where players cannot discriminate in their actions between other players, i.e., one action (efficient or inefficient) has to be chosen towards all other players. While this set-up is in line with prominent theoretical literature on neighborhood choice^30,44^, strategic situations are perceivable where discrimination in actions between players is possible. There are two reasons why we have chosen for our environment. First, when allowing for discrimination in actions, the 6-person stag-hunt game we are analyzing would collapse to a number of 2-player stag-hunt games played simultaneously. This changes the strategic situation in a way that makes it less interesting, in our view. Specifically, when discrimination in actions is possible it suffices that the probability of *some* of the other players playing efficiently is high enough, to make playing efficiently a best response against *each* of these players. Thus, it is relatively likely that efficient outcomes emerge in some of the 2-player stag-hunt games. In contrast, without discrimination in actions, playing the efficient action is a best response only if *all* other five players also play the efficient action, otherwise it is better to choose the inefficient action against all other players. Thus, the efficient action should be played only when the joint probability of *all* others playing efficiently too is very high, which makes it much more likely to observe all players playing inefficiently. Therefore, we consider the variant without discrimination in action as the more challenging one.

Second, the set-up of no discrimination in action reflects interesting interaction patterns in the field at an individual as well as more aggregate level. For instance, when work is co-authored an individual author cannot discriminate in her contribution between the individual co-authors. Neighborhood choice here would mean that one can decide whether or not to work with a specific co-author in future projects. A similar reasoning can be applied to other forms of team work. On a country level, reducing CO$_2$-emissions by introducing a carbon tax is another example. If not all countries implement the tax, CO$_2$-intensive production will shift to those countries that do not have such a tax. In that case, the total emission of CO$_2$ will not change significantly, while the introducing countries will have great economic disadvantages and ‘defecting’ countries reap the economic benefits from the shift. Here, neighborhood choice may be implemented by means of economic or political sanctions.

Inclusion of efficiently playing agents and exclusion of inefficiently playing agents has the flavor of reward and punishment, respectively, two mechanisms much investigated in cooperation problems^43,45–49^. However, for coordination games, very little is known about the effect of punishment^50,51^ and, to our knowledge, nothing about the effect of reward. We do not observe a significant effect of including efficiently playing agents into one’s neighborhood, which contrasts with the positive effect of reward identified in some cooperation problems^49^. A possible reason for this discrepancy is that the reward mechanism tested in cooperation problems is commonly adding ‘manna from heaven’ to the environment and is thus not only rewarding but also efficiency enhancing. This additional channel is not available with inclusion in our environment. The exclusion of inefficiently playing agents sorts two effects. First, regarding the excluded agent it entails a costly punishment effect because it is expensive and hurts the excluded agent. The literature on cooperation games has shown that punishment can be an effective mean to increase cooperation. The punishment aspect entailed in exclusion could be a reason behind the effectiveness of neighborhood choice we observe. Second, however, exclusion also reduces strategic uncertainty as it allows efficiently playing agents to ‘remove’ inefficiently acting agents from their neighborhood. Future research could investigate the relative importance of these two channels for enhancing efficient coordination through neighborhood choice.

In summary, we provide empirical evidence on the effect of neighborhood choice on the efficiency of coordination in stag-hunt games. Theoretical work on endogenous interaction choice in coordination games is ambiguous on how neighborhood choice may affect the efficiency of coordination. In our experiment neighborhood choice has a clear and substantial positive effect which gets stronger over time. This suggests that in order to achieve efficient outcomes social institutions should be build in a way that gives individuals sufficient levy in the choice of their interaction neighborhood.

## Methods

All experiments were conducted in the Behavioral and Experimental Economics laboratory (BEElab) at Maastricht University. The experiment was reviewed and approved by the BEElab board and all methods were performed in accordance with the relevant guidelines and regulations. Potential subjects were recruited for the BEElab subject pool through email announcements and announcements on students’ intranet. Upon subscribing subjects provided informed consent. All experiment sessions were conducted in the BEElab and all subjects were recruited from the BEElab subject pool. In total $n = 108$ subjects (mean age $= 22.7$, women $=43\%$; for more detailed information on subjects’ characteristics and a comparison between treatments see the SI) participated, randomly and equally distributed over 18 sessions (9 sessions of Imposed, 9 sessions of Free). All subjects were students of Maastricht University and each participated in only one session and none had participated in a similar experiment before.

During the experiment, subjects received computerized and written instructions which they could study at their own pace. Additionally they could ask questions privately. Only questions about the instructions were allowed. No answer was given if it could have influenced the individuals’ expectations or strategy choice. The experiment instructions avoided suggestive labels like ‘efficient’ or ‘safe’ actions. Instead neutral language was used and different actions in the stag-hunt game were indicated by the colors blue and green. After having read the instructions all subjects passed comprehension questions checking for correct understanding of the experimental procedures and financial incentives. In line with the norm in experimental economics the experiment did not involve deception. On average, subjects earned €21,–, with individual earnings depending on their decisions in the experiment. In addition they received a €5,– show-up fee. At the end of the session subjects were paid their earnings in private.

Each session lasted for about two hours and consisted of five parts. Subjects received information and instructions for a part only after the previous part was finished. In Part 1 we elicited social preferences using an adopted version of the social value orientation test^52^, Part 2 comprised the 30 rounds of stag-hunt games described above, Part 3 consisted of another 30 rounds of stag-hunt games after reshuffling subjects into new groups of six, Part 4 was a Holt-Laury risk attitudes elicitation task^53^, and in Part 5 subjects answered questions about some basic demographics (for a detailed description of the different parts see the SI). The coordination outcomes in Part 3 are similar to those in Part 2 and reported in the SI. In the SI we also provide tests showing that subjects did not differ significantly between treatments in their social preferences, risk attitudes, age, gender, nationality, study year or field of study.

Reported tests are two-sided, unless stated otherwise. Sample sizes for the study were not based on an explicit power analysis due to a lack of directly comparable experiments to base the power analysis on. Unless stated otherwise, the analyses reported in the main text are based on regression analyses with standard errors corrected for data dependency within interaction group (except for round 1 where individual data are treated as independent). The SI report more conservative analyses using non-parametric tests based on group averages as the (strictly independent) unit of observations. These analyses confirm all the main results regarding the (in)efficiency of action choices, the effect of inclusion and exclusion, and the differences in welfare gains between treatments. Our bar graphs do not show standard error bars because they are not informative given the use of logit and Tobit regressions and non-parametric statistical analyses. Outcome frequencies for each individual group are reported in the SI.

Data collection and analyses were not performed blind to the treatments of the experiment. No data were excluded from the reported analyses.

## Supplementary Information

Supplementary Information.

## Data Availability

Upon publication of the manuscript, all data collected for this study will be made freely available.
