# Supplementary material for: Free neighborhood choice boosts socially optimal outcomes in stag-hunt coordination problem
Source: Sci Rep. 2021 Apr 8;11:7745. doi: 10.1038/s41598-021-87019-y (PMC8032720; doi:10.1038/s41598-021-87019-y)
Supplement: Supplementary file 1 — Supplementary Information. [file 41598_2021_87019_MOESM1_ESM.pdf]

Supplementary Information (SI) for
"Free neighborhood choice boosts socially optimal outcomes in stag-hunt coordination problem"

by **Arno Riedl, Ingrid M.T. Rohde, Martin Strobel**

Contents

A Statistical analyzes – Observable characteristics **2**

B Statistical analyzes – Logit regressions **3**

C Statistical analyzes – Non-parametric tests **13**

D Outcomes of individual groups **16**

E Frequency of outcomes in Part 3 **21**

F Experiment design **23**

G Experiment instructions **25**

A Statistical analyzes – Observable characteristics

Table A.1. Subjects' observable characteristics and differences between treatments

Characteristic	Treatments IMPOSED	FREE	Diff. treatments *p*-value
Age	23.2	22.1	0.1141[1]
	(3.29)	(2.41)	
Study year	2.8	2.4	0.1137[1]
	(1.54)	(1.38)	
Risk attitudes	6.0	5.8	0.6216[1]
	(1.86)	1.79	
Social preferences	0.52	0.62	0.2339[1]
	(1.018)	(0.849)	
Gender			0.560[2]
female	25	21	
male	29	33	
Nationality			0.209[2]
German	16	26	
Dutch	18	12	
Chinese	6	3	
other	14	13	
Field of study			0.307[2]
Business or Economics	33	39	
other	21	15	

Note: Means (standard deviations) of age, study year, risk attitudes, and social preferences; absolute frequencies of gender, nationality, and field of study; [1] ... 2-sided Mann-Whitney test, [2] ... 2-sided Fisher's exact test.

B Statistical analyzes – Logit regressions

Table B.1. Comparison of frequency of outcomes between FREE and IMPOSED (logit regressions, all rounds)

	Efficient Coordination		Inefficient Coordination		Miscoordination	
	coef./std. err.	p -value	coef./std. err.	p -value	coef./std. err.	p -value
FREE	1.628*	0.039	-2.125*	0.025	-0.435	0.203
	(0.787)		(0.946)		(0.342)	
Const.	-0.705	0.255	0.162	0.773	-1.911***	0.000
	(0.619)		(0.563)		(0.293)	
Wald χ^2	4.27		5.05		1.62	
# clusters	18		18		18	
N	8100		8100		8100	

Note: Reference treatment is IMPOSED.

Standard errors in parentheses adjusted for 18 clusters (i.e., group of five interacting dyads).

* $p < 0.05$, ** $p < 0.01$, *** $p < 0.001$

Table B.2. Comparison of frequency of outcomes between Free and Imposed (logit regressions, round 1)

	Efficient Coordination		Inefficient Coordination		Miscoordination	
	coef./std. err.	p -value	coef./std. err.	p -value	coef./std. err.	p -value
Free	0.352	0.612	-1.068	0.064	-0.314	0.431
	(0.694)		(0.576)		(0.398)	
Const.	-0.727	0.139	-1.089*	0.017	-0.314	0.223
	(0.492)		(0.457)		(0.258)	
Wald χ^2	0.26		3.44		0.62	
N	270		270		270	

Note: Reference treatment is Imposed.

Standard errors in parentheses (unadjusted for clusters because in round 1 individual data are independent).

* $p < 0.05$, ** $p < 0.01$, *** $p < 0.001$

Table B.3. Comparison of frequency of outcomes between Free and Imposed (logit regressions, rounds 1-10)

	Efficient Coordination		Inefficient Coordination		Miscoordination	
	coef./std. err.	p -value	coef./std. err.	p -value	coef./std. err.	p -value
Free	0.971	0.204	-1.466	0.061	-0.490	0.210
	(0.765)		(0.783)		(0.390)	
Const.	-0.730	0.197	-0.226	0.660	-1.202***	0.000
	(0.566)		(0.513)		(0.288)	
Wald χ^2	1.61		3.50		1.57	
# clusters	18		18		18	
N	2700		2700		2700	

Note: Reference treatment is Imposed.

Standard errors in parentheses adjusted for 18 clusters (i.e., group of five interacting dyads).

* $p < 0.05$, ** $p < 0.01$, *** $p < 0.001$

Table B.4. Comparison of frequency of outcomes between FREE and IMPOSED (logit regressions, rounds 11-20)

	Efficient Coordination		Inefficient Coordination		Miscoordination	
	coef./std. err.	p -value	coef./std. err.	p -value	coef./std. err.	p -value
FREE	1.934*	0.031	-2.510*	0.036	-0.773	0.225
	(0.894)		(1.200)		(0.637)	
Const.	-0.575	0.369	0.137	0.820	-2.133***	0.000
	(0.641)		(0.599)		(0.516)	
Wald χ^2	4.68		4.37		1.47	
# clusters	18		18		18	
N	2700		2700		2700	

Note: Reference treatment is IMPOSED.

Standard errors in parentheses adjusted for 18 clusters (i.e., group of five interacting dyads).

* $p < 0.05$, ** $p < 0.01$, *** $p < 0.001$

Table B.5. Comparison of frequency of outcomes between FREE and IMPOSED (logit regressions, rounds 21-30)

	Efficient Coordination		Inefficient Coordination		Miscoordination	
	coef./std. err.	p -value	coef./std. err.	p -value	coef./std. err.	p -value
FREE	2.147*	0.020	-2.502*	0.032	0.105	0.879
	(0.925)		(1.168)		(0.687)	
Const.	-0.816	0.240	0.591	0.389	-2.952***	0.000
	(0.694)		(0.687)		(0.567)	
Wald χ^2	5.39		4.59		0.02	
# clusters	18		18		18	
N	2700		2700		2700	

Note: Reference treatment is IMPOSED.

Standard errors in parentheses adjusted for 18 clusters (i.e., group of five interacting dyads).

* $p < 0.05$, ** $p < 0.01$, *** $p < 0.001$

Table B.6. Comparison of inclusion frequencies conditional on other's action in $t-1$ and own action in t (logit regression)

	Likelihood of inclsuion in t	
	coeff./std. err.	p -value
(1) other's inefficient & own inefficient	0.191	0.570
	(0.335)	
(2) other's efficient & own inefficient	0.885**	0.001
	(0.270)	
(3) other's inefficient & own efficient	-2.686***	0.000
	(0.600)	
Const.	0.955**	0.001
	(0.296)	
(1) vs (2)	0.012	
Wald chi2	50.91	
# clusters	7	
N	592	

Note: Reference case is 'other's efficient & own efficient'.

Standard errors in parantheses adjusted for 7 clusters (i.e., group of five interacting dyads). There are only 7 clusters because in two groups no instances of inclusion occured.

* $p < 0.05$, ** $p < 0.01$, *** $p < 0.001$

Table B.7. Comparison of exclusion frequencies conditional on other's action in $t-1$ and own action in t (logit regression)

	Likelihood of exclsuion in t	
	coeff./std. err.	p -value
other's inefficient & own efficient	5.038***	0.000
	(0.841)	
Const.	-6.240***	0.000
	(0.788)	
Wald chi2	35.9	
# clusters	9	
N	5970	

Note: Reference case is 'other's efficient & own efficient'.
Standard errors in parantheses adjusted for 9 clusters (i.e., group of five interacting dyads). Instances where a subject chooses the inefficient action and excludes another subject happen in less than 0.4% of the cases; therefore, these observations are omitted.
* $p < 0.05$, ** $p < 0.01$, *** $p < 0.001$

Table B.8. Inertia: likelihood to choose an efficient action in $t+1$ after having chosen an (in)efficient action in $t-1$ (logit regression)

	Likelihood of efficient action in $t+1$	
	coeff./std. err.	p -value
efficient action in $t-1$	4.432***	0.000
	(0.608)	
Const.	-1.346*	0.045
	(0.671)	
Wald chi2	53.1	
# clusters	9	
N	7560	

Note: Reference case is 'inefficient action in $t-1$'.
Standard errors in parantheses adjusted for 9 clusters (i.e., group of five interacting dyads).
* $p < 0.05$, ** $p < 0.01$, *** $p < 0.001$

Table B.9. Comparison of effects of (no) inclusion in t on likelihood to choose the efficient action in $t+1$ (logit regression)

	Likelihood of efficient action in $t+1$	
	coeff./std. err.	p -value
(1) no inclusion in t after inefficient action in $t-1$	-2.491***	0.000
	(0.245)	
(2) inclusion in t after inefficient action in $t-1$	-2.583*	0.012
	(1.027)	
(3) no inclusion in t after efficient action in $t-1$	-0.268	0.345
	(0.283)	
Const.	2.072***	0.000
	(0.500)	
(1) vs (2)	0.928	
Wald chi2	186.6	
# clusters	7	
N	586	

Note: Reference case is 'inclusion in t after efficient action in $t-1$'.

Standard errors in parentheses adjusted for 7 clusters (i.e., group of five interacting dyads). There are only 7 clusters because in two groups no instances of inclusion occured.

* $p < 0.05$, ** $p < 0.01$, *** $p < 0.001$

Table B.10. Comparison of effects of (no) exclusion in t on likelihood to choose the efficient action in $t+1$ (logit regression)

	Likelihood of efficient action in $t+1$	
	coeff./std. err.	p -value
(1) exclusion in t after efficient action in $t-1$	3.642***	0.000
	(0.286)	
(2) no exclusion in t after efficient action in $t-1$	4.919***	0.000
	(0.637)	
(3) exclusion in t after inefficient action in $t-1$	1.750***	0.000
	(0.453)	
Const.	-1.696*	0.015
	(0.695)	
(1) vs (2)	0.031	
Wald chi2	191.7	
# clusters	9	
N	6974	

Note: Reference case is 'no exclusion in t after inefficient action in $t-1$'.

Standard errors in parentheses adjusted for 9 clusters (i.e., group of five interacting dyads).

* $p < 0.05$, ** $p < 0.01$, *** $p < 0.001$

Table B.11. Difference in welfare gain between treatments across rounds 1-10 (Tobit regression)

	Welfare gain	
	coeff./std. err.	p -value
Free	0.182	0.819
	(0.796)	
Const.	0.575	0.243
	(0.493)	
sigma	2.373***	0.000
	(0.488)	
p(Free = 0)	0.313	
F	0.052	
Prob > F	0.819	
# clusters	18	
N	5400	

Note: Reference treatment is Imposed.

Standard errors in parentheses adjusted for 18 clusters

(i.e., group of five interacting dyads);

dependent variable is left-censored at -3.75 and right-censored at 1.

* $p < 0.05$, ** $p < 0.01$, *** $p < 0.001$

Table B.12. Difference in welfare gain between treatments across rounds 11-20 (Tobit regression)

	Welfare gain	
	coeff./std. err.	p -value
Free	1.431	0.075
	(0.803)	
Const.	0.825	0.178
	(0.612)	
sigma	2.203***	0.000
	(0.628)	
$p(\text{Free}) = 0$	0.013	
F	3.174	
# clusters	18	
N	5400	

Note: Reference treatment is Imposed. Standard errors in parentheses adjusted for 18 clusters (i.e., group of five interacting dyads); dependent variable is left-censored at -3.75 and right-censored at 1.

* $p < 0.05$, ** $p < 0.01$, *** $p < 0.001$

Table B.13. Difference in welfare gain between treatments across rounds 21-30 (Tobit regression)

	Welfare gain	
	coeff./std. err.	p -value
FREE	1.283*	0.020
	(0.550)	
Const.	0.559	0.165
	(0.402)	
sigma	1.493***	0.001
	(0.433)	
$p(\text{FREE}) = 0$	0.004	
F	5.434	
# clusters	18	
N	5400	

Note: Reference treatment is IMPOSED.
Standard errors in parentheses adjusted for 18 clusters (i.e., group of five interacting dyads); dependent variable is left-censored at -3.75 and right-censored at 1.
* $p < 0.05$, ** $p < 0.01$, *** $p < 0.001$

C Statistical analyzes – Non-parametric tests

Non-parametric tests for the main results reported in the main text using aggregate group outocomes as unit of observations.

Table C.1. Frequency of outcomes

Frequency of outcomes (all rounds)		
miscoordination		
Imposed	13%	$p = 0.3765$ [a]
Free	9%	$n = 18$
inefficient coordination		
Imposed	54%	$p = 0.1215$
Free	12%	$n = 18$
efficient coordination		
Imposed	33%	$p = 0.0574$
Free	72%	$n = 18$
Frequency of outcomes (round 1)		
miscoordination		
Imposed	42%	$p = 0.3432$
Free	35%	$n = 18$
inefficient coordination		
Imposed	25%	$p = 0.2994$
Free	10%	$n = 18$
efficient coordination		
Imposed	33%	$p = 0.6229$
Free	41%	$n = 18$

[a] All tests are 2-sided Mann-Whitney tests.

Table C.2. Inclusion and exclusion

Inclusion rate in t		
own inefficient action in t and ...		
others inefficient action in $t-1$	76%	$p = 0.5775$[a]
others efficient action in $t-1$	86%	$n = n = 4$
own efficient action in t and ...		
others inefficient action in $t-1$	15%	$p = 0.0464$
others efficient action in $t-1$	72%	$n = 6$
Exclusion rate in t		
own inefficient action in t and ...		
others inefficient action in $t-1$	0%	$p = 0.0854$
others efficient action in $t-1$	2%	$n = 8$
own efficient action in t and ...		
others inefficient action in $t-1$	23%	$p = 0.0140$
others efficient action in $t-1$	$< 0.2\%$	$n = 8$

[a] All tests are 2-sided Wicooxon signed-rank tests. $n < 9$ means that the explored situations did not happen in some groups.

Table C.3. Inertia and the effect of inclusion and exclusion

Rate of efficient action in $t+1$		
after inefficient action in $t-1$	21%	$p = 0.0173$[a]
after efficient action in $t-1$	96%	$n = 8$
after inefficient action in $t-1$ and ...		
no inclusion in t	40%	$p = 0.3883$
inclusion in t	38%	$n = 6$
after efficient action in $t-1$ and ...		
no inclusion in t	86%	$p = 0.6662$
inclusion in t	89%	$n = 6$
after inefficient action in $t-1$ and ...		
no exclusion in t	16%	$p = 0.1493$
exclusion in t	51%	$n = 7$
after efficient action in $t-1$ and ...		
no exclusion in t	96%	$p = 0.7150$
exclusion in t	88%	$n = 4$

[a] All tests are 2-sided Wicooxon signed-rank tests. $n < 9$ means that the explored situations did not happen in some groups.

Table C.4. Welfare gains

Early in game (rounds 1-10)		
Imposed	0.01	$p = 0.8597$[a]
Free	−0.14	$n = 18$
Intermediate in game (rounds 11-20)		
Imposed	0.21	$p = 0.4234$[a]
Free	0.47	$n = 18$
Late in game (rounds 21-30)		
Imposed	0.24	$p = 0.0419$[a]
Free	0.62	$n = 18$

[a] All tests are 2-sided Mann-Whitney tests.

D Outcomes of individual groups

All rounds

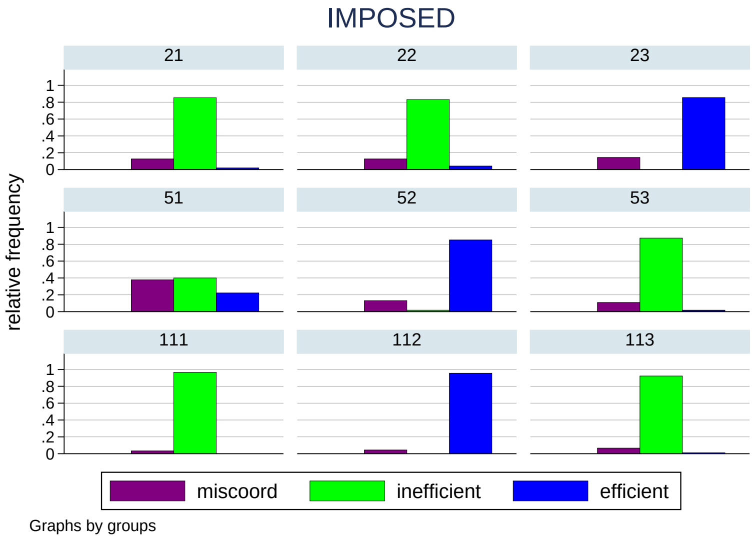

Figure D.1. Frequency of the different outcomes in each group in imposed neighborhoods (Imposed) aggregated over all rounds.

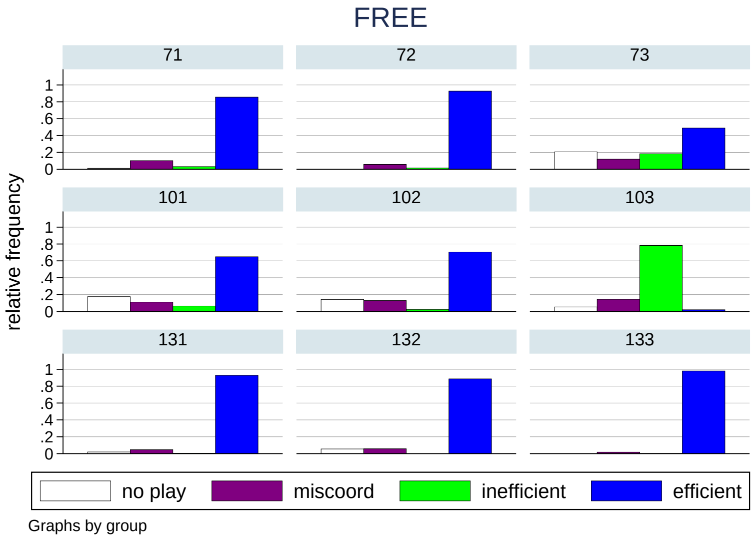

Figure D.2. Frequency of the different outcomes in each group in free neighborhood choice (Free) aggregated over all rounds.

Round 1

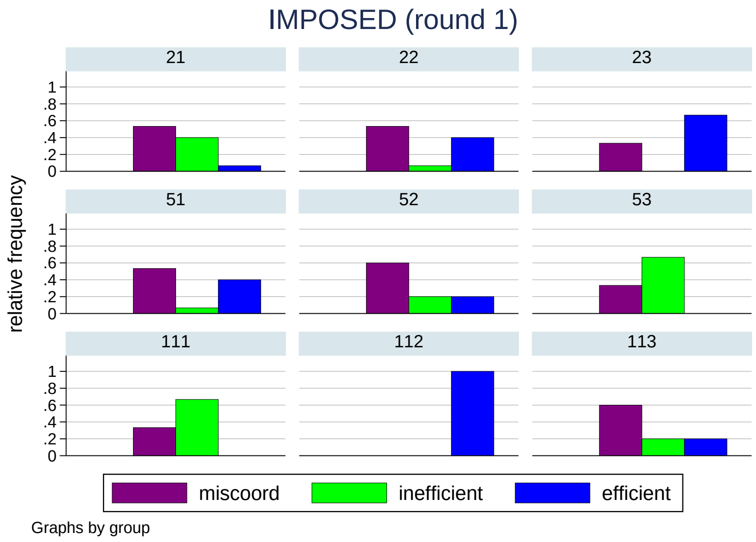

Figure D.3. Frequency of the different outcomes in each group in imposed neighborhoods (Imposed) in round 1.

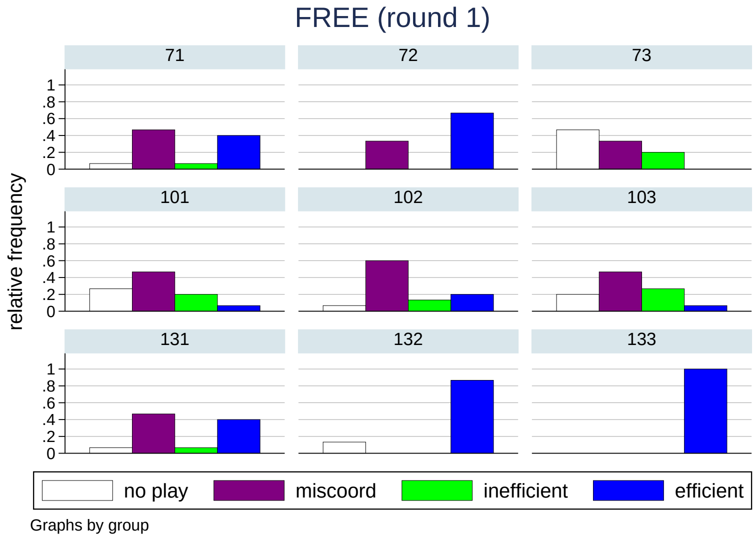

Figure D.4. Frequency of the different outcomes in each group in free neighborhood choice (Free) in round 1.

Rounds 1–10

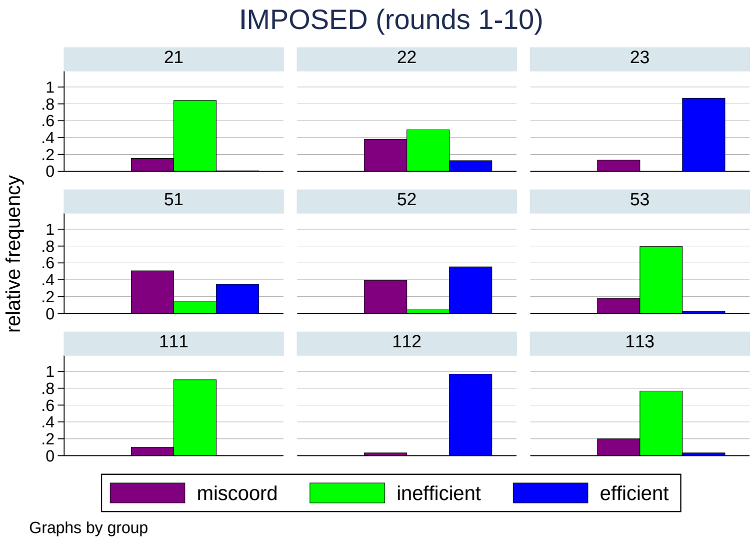

Figure D.5. Frequency of the different outcomes in each group in imposed neighborhoods (Imposed) in rounds 1–10.

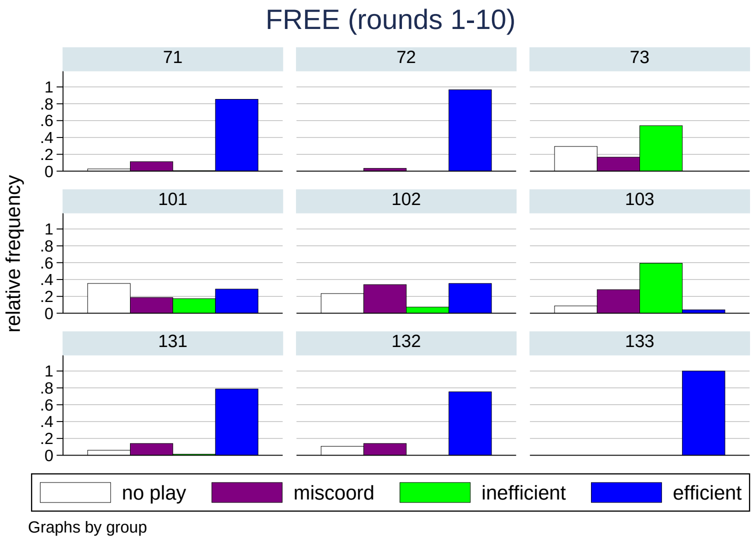

Figure D.6. Frequency of the different outcomes in each group in free neighborhood choice (Free) in rounds 1–10.

Rounds 11–20

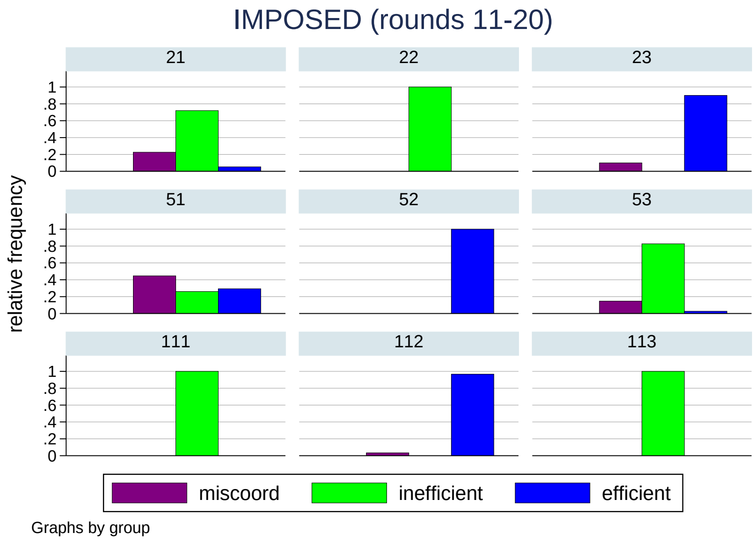

Figure D.7. Frequency of the different outcomes in each group in imposed neighborhoods (IMPOSED) in rounds 11–20.

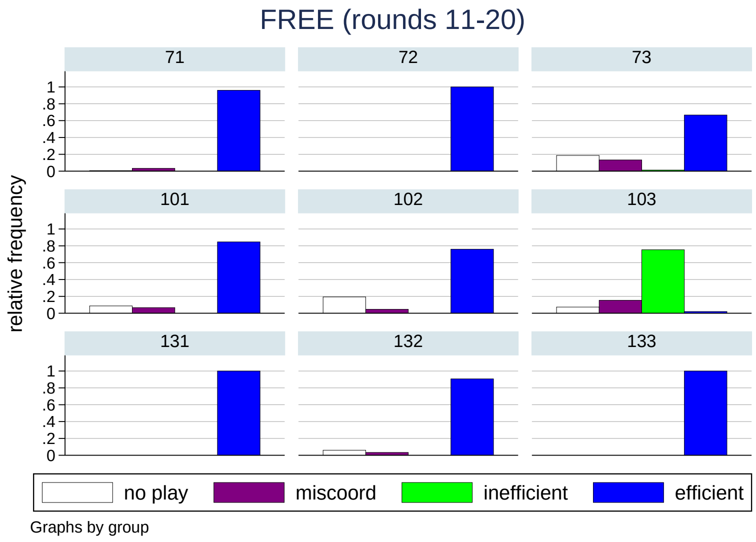

Figure D.8. Frequency of the different outcomes in each group in free neighborhood choice (FREE) in rounds 11–20.

Rounds 21–30

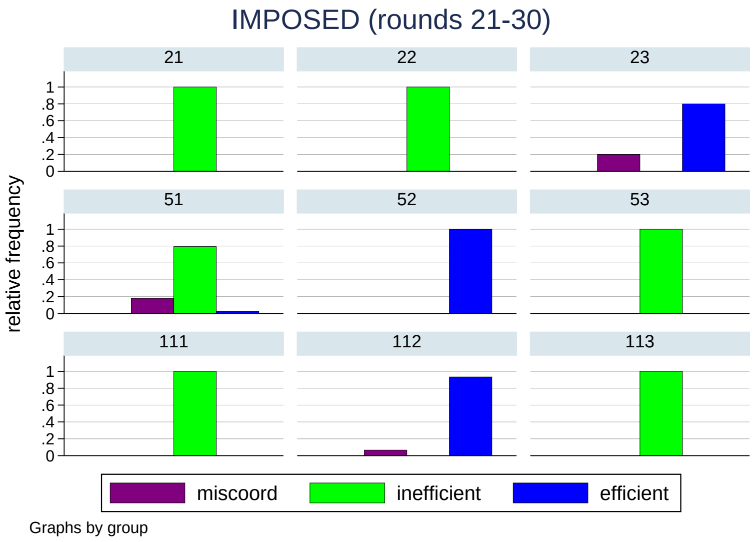

Figure D.9. Frequency of the different outcomes in each group in imposed neighborhoods (IMPOSED) in rounds 21–30.

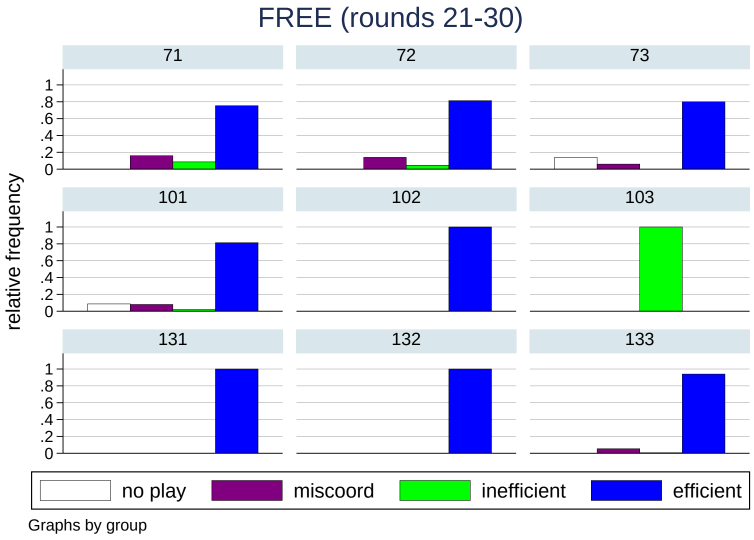

Figure D.10. Frequency of the different outcomes in each group in free neighborhood choice (FREE) in rounds 21–30.

E Frequency of outcomes in Part 3

Part 3 consisted of another 30 rounds of stag-hunt games after reshuffling participants into new groups of six. Here we report the frequency of outcomes corresponding to those reported in the main text. Comparison of the figure here with those in the main text shows that overall efficient coordination is more prevalent in both Imposed and Free, but that the differences between the treatments remain distinct.

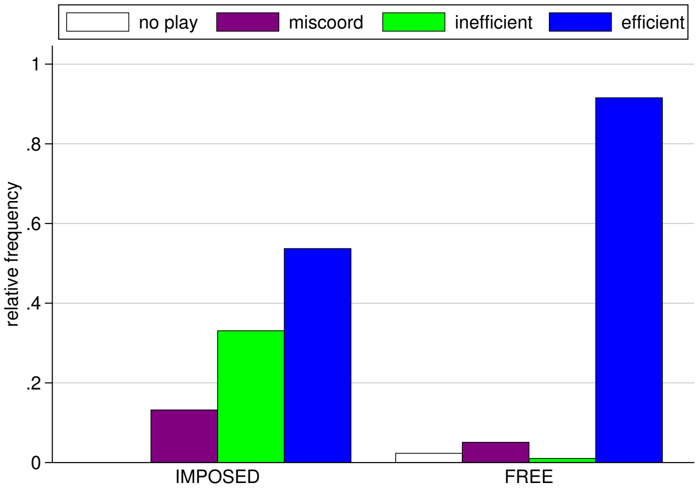

Figure E.1. Frequency of the different outcomes in Part 3 in imposed neighborhoods (Imposed) and free neighborhood choice (Free) aggregated over all rounds. Inefficient coordination (green) is relatively frequent in Imposed but basically absent in Free, whereas efficient coordination (blue) is much more frequent in Free than in Imposed. Miscoordination (purple) is slightly more frequent in Imposed than in Free. No-play (white) can only occur in Free and is extremely infrequent.

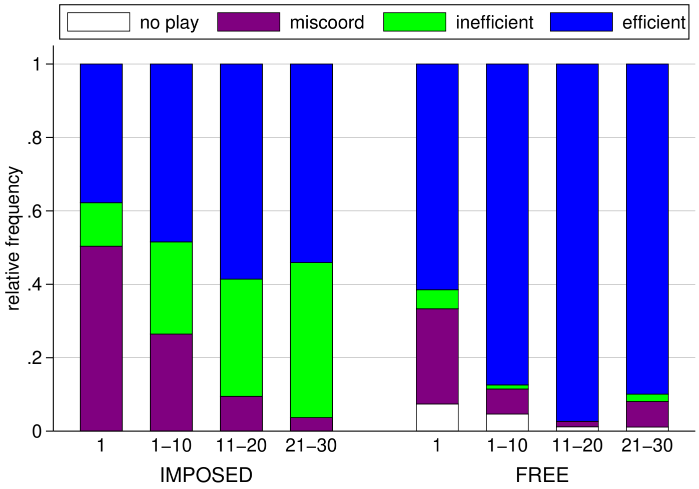

Figure E.2. Frequency of the different outcomes in Part 3 in Imposed and Free in round 1 and aggregated over rounds 1-10, 11-20, and 21-30. Already in round 1 is the frequency of efficient coordination higher in Free than in Imposed. Over rounds the difference between treatments increases as inefficient coordination (green) becomes more frequent in Imposed whereas efficient coordination (blue) becomes more frequent in Free. Miscoordination (purple) decreases in both treatments. No-play (white), only possible in Free, disappears over time.

F Experiment design

All experiments were conducted in the Behavioral and Experimental Economics laboratory (BEElab) at Maastricht University. Subjects were recruited through email announcements and announcements on students' intranet. In total 108 subjects participated, randomly and equally distributed over 18 sessions (9 sessions of Imposed, 9 sessions of Free). All subjects were students of Maastricht University and each participated in only one session and none had participated in a similar experiment before. During the experiment, subjects received computerized and written instructions which they could study at their own pace. Additionally they could ask questions privately. Only questions about the instructions were allowed. No answer was given if it could have influenced the individuals' expectations or strategy choice. On average, subjects earned €21,–, with individual earnings depending on their decisions in the experiment. In addition they received a €5,– show-up fee. At the end of the session subjects were paid their earnings in private. Each session took approximately two hours.

Each session consisted of five parts. Subjects received information and instructions for a part only after the previous part was finished. Part 1 consisted of an adopted incentivized version of the social value orientation test [3], Part 2 comprised the 30 rounds of stag-hunt games reported in the main text, Part 3 consisted of another 30 rounds of stag-hunt games after reshuffling subjects into new groups of six, in Part 4 subjects performed an incentivized risk preferences elicitation task à la Holt and Laury [1], and in Part 5 subjects answered questions about some basic demographics. We now describe the different parts in some detail.

In Part 1 we measured subjects' attitudes towards others' welfare and efficiency concerns. For that we developed an extended version of the circle test [2] which is based on the social value orientation test [3]. Specifically, subjects were asked to select a point on a circle and on two differently shaped ellipses. Each point on the circle or ellipse, corresponded to a certain allocation of points to the person making the decision (S) and to another anonymous individual (O).The horizontal dimension represented the allocation for oneself whereas the vertical dimension determined the allocation for the other person. Each point on the circle and ellipses yielded a measure to quantify people's disposition to others and their disposition to efficiency concerns. Importantly, for each of the three decisions in this part subjects were matched into new pairs and pairs matched in this part did not interact in any other parts. Subjects were informed about that and did not receive and any feedback from the circle and ellipse tests until the end of the experiment.

By choosing an appropriate coordinate system, the circle and the two ellipses can be described by the following equations. Circle: $\frac{S^2}{2^2} + \frac{O^2}{2^2} = 1$, Ellipse 1: $\frac{S^2}{1^2} + \frac{O^2}{3^2} = 1$, Ellipse 2: $\frac{S^2}{3^2} + \frac{O^2}{1^2} = 1$. The maximum number of points a subject could allocate to her-/himself and the other anonymous subject were as follows. Circle: $(S, O) = (816, 816)$, Ellipse 1: $(S, O) = (1224, 408)$, Ellipse 2: $(S, O) = (408, 1224)$. For screenshots of how these tasks were presented to the subjects, see the experiment instructions (Section G). To calculate each subject's attitude towards pro-sociality and efficiency we assumed the utility specification $u_i(x_i, x_j) = x_i + \theta x_j$, where x_i (x_j) is the amount the subject allocated to her-/himself. We then calculated for each of the three tasks the corresponding θ and used the average of the three θ's as our measure for pro-social and efficiency attitudes (see Table A.1).

In Part 2 subjects played the the stag-hunt games described in the main text. In each session the 18 subjects were randomly divided into three independent groups of 6 subjects. To assure anonymity, in the experiment subjects were referred to themselves as "Me" and the other members of their group

were labeled with the letters "A","B", "C", "D", and "E". After reading the instructions all subjects had to correctly answer some comprehension questions. They then played 30 rounds of the stag-hunt game (see Fig. F.1) either with all other members in their group (treatment IMPOSED) or in an interaction neighborhood that depended on the interaction proposals made in the stag-hunt game (treatment FREE). Two subjects were interacting with each other, i.e., were in the same interaction neighborhood, only when both had proposed to interact with each other. When two subjects did not interact they earned zero because no stag-hunt game was played between them. At the beginning of each round, subjects could

	Blue	*Green*
Blue	95,95	5,90
Green	90,5	75,75

Figure F.1. Stag-hunt game payoff matrix

update their action choices in the stag-hunt game and, in treatment FREE, their interaction proposals. In the latter treatment choices in the stag-hunt game and interaction proposals were made simultaneously. In each round in Part 2, subjects could access all information on the past actions and interaction proposals chosen by all subjects in their group. In addition they were informed about their own earnings in each round and accumulated over all preceding rounds. For screen-shots subjects saw when making their decisions please see the main text and the instructions (Section G).

At the beginning of Part 3 subjects were randomly reshuffled into new groups of six. At this point they were also informed about this change and that they will interact in the same game as before for another 30 rounds.

Part 4 consisted of a risk preferences elicitation task based on the method developed by Holt and Laury[1]. In this task subjects saw a table of ten paired lotteries ('Option A' and 'Option B') in which they had to determine for each pair which lottery they prefer. Option A outcomes were always 2000 points and 1600 points, respectively, and Option B Outcomes B were always 3850 and 100 points, respectively. What changed across paired lotteries were the probabilities with which the outcomes occurred. In Option A the probability of 2000 (1600) increased from 10% to 100% (decreased from 90% to 0%) and in Option B the probability of 3850 (100) increased from 10% to 100% (decreased from 90% to 0%). See the experiment instructions for a screen-shot of the paired lotteries as presented to the subjects (Section G). A risk neutral subject would switch from Option A to Option B when the expected value of the latter is larger then of the former. As the spread of Option B is larger than the spread of Option A a risk averse subject would switch to Option B at a later point. Thus the later a subject switches from Option A to Option B the more risk averse (less risk seeking) the subject was. We use the switching point as a measure of subjects' risk attitudes.

Part 5 consisted of a short questionnaire where subjects answered a number of demographic questions.

Thereafter subjects were paid out in private the sum of their earnings in Parts 1 to 4. For Part 1 a subject earned according to her/his own choices in the circle and two ellipses plus the amounts assigned to them in these three tasks by three randomly chosen other participants. Part 2 and Part 3 earnings consisted of the sum of earnings across the 30 rounds in each part. In Part 4, after subjects had made their decisions one of the 10 paired lottery choices was randomly, with equal likelihood, selected to be payoff relevant. Thereafter, the lottery of the chosen option was run and the outcome added to the subject's earnings.

G Experiment instructions

Introduction

Welcome to this experiment on decision-making. In this experiment you can earn money. How much you earn depends on your decisions and the decisions of other participants.

The experiment consists of four independent parts. In each part you can earn points. Your earnings in each part are independent of your earnings in the other part. At the end of the experiment you get paid your earned points privately in cash, according to the exchange rate:

100 points = 8 eurocent

First you receive the instructions for the first part. You will get the instructions for the following part only after the preceding part finished. At the end of the experiment you will be asked to fill in a short questionnaire. Thereafter you will be paid your earnings.

During the whole experiment, you are not allowed to communicate with other participants in any other way than specified in the instructions.

If you have a question, please raise your hand. We will then come to you to answer it.

Instructions part I

In part I of the experiment you are asked to make three decisions. These decisions concern the assignment of an amount (of points) to yourself and an amount to another, arbitrarily chosen participant.

You received a print-out of the computer screen for part I from us. Please take this print-out in front of you. We shall explain your choice options with the help of the print-out.

On the first screen you see a **circle**. The second and third screen will show **ellipses**. By choosing a point on the circle and ellipses you decide about the assignment of an amount of points to yourself and an amount of points to another participant in this experiment. This other participant will be chosen completely randomly by the computer.

Note: In each of these three decision rounds you will be linked to another participant. Furthermore, you will never meet a person who has already made, is making or who will, in the future, make a decision that affects your earnings.

Each point on the circle and ellipses represents an amount that is added to your earnings (+) or is subtracted from your earnings (−) and an amount that is added to the earnings of the other participant (+) or is subtracted from his or her earnings (−). Hence, with your decision you can increase or decrease your earnings and the earnings of the other participant.

You can click on any point of the circle/ ellipse with the help of your mouse. You will then see an arrow that shows you the decision you made. Additionally, you will see the corresponding amounts (points) in the window 'decision', at the right of the circle/ ellipse. In the **'for you'** field you will see the amount that you assigned to yourself, and in the **'for the other'** field the amount you assigned to the other participant. To change your decision you can click on another point of the circle. You may also use the buttons below the circle. With these buttons you can refine your decision: you can move the arrow a little bit in the clockwise direction (left button) or in the counter-clockwise direction (right button). If you are satisfied with your decision you have to confirm it by clicking on the button **'Confirm'**. An **'Are you sure?'** window will appear then. Click on 'yes' if you do not want to change your choice anymore.

For each circle/ ellipse your earnings will depend on your own decision ***and*** the decision of one other, randomly chosen, participant you are paired with. This other participant has to make a similar decision as you made. **Note:** In each of these three decision rounds you will be linked to another participant. Furthermore, you will ***never*** meet a person who has already made, is making or who will, in the future, make a decision that affects your earnings. Your earnings in this part of the experiment are equal to the sum of the amounts you assign to yourself and the amounts that the three randomly chosen other participants assign to you.

Note: these amounts can be positive but also negative. In the latter case the amount will be subtracted from the earnings of the respective recipient.

During the whole experiment, you will not receive any information about the decisions of the other participants you are paired with. Only after the end of the experiment you will get informed about the amount these other participants have assigned to you. Similarly, the other participants will get informed about the amount you assign to her or him only after the end of the experiment.

Part I of the experiment will begin shortly. **There is no practice round.**

If you have any questions now, please raise your hand. If you do not have any questions any more, please click on **'READY'**.

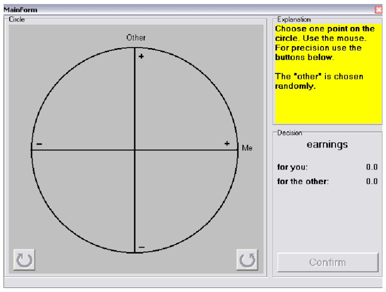

(a) Circle

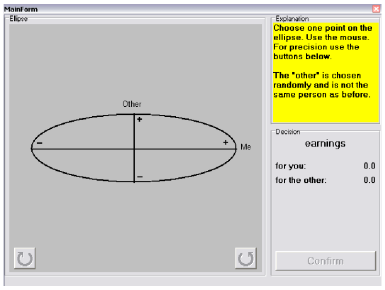

(b) Ellipse 1

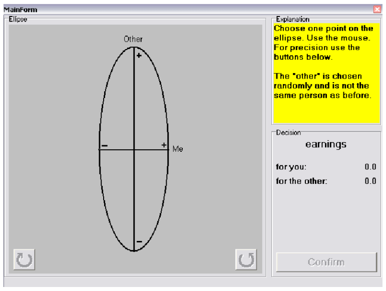

(c) Ellipse 2

Figure G.1. Computer screens circle and ellipse tasks

In the following we provide the instructions for Part 2. First, for treatment Imposed followed by the instructions for treatment Free. In the original instructions treatment names were never mentioned. They are mentioned here for convenience of the reader.

Instructions part II (treatment Imposed)

In this part of the experiment every participant is in a **group of six** with five other participants. Before part II begins, these groups of six will be formed arbitrarily with the help of the computer. The group you are in will not change during this part of the experiment. You will not get information about the identity of the persons in your group, neither during the experiment, nor after the experiment. Other participants will also not receive any information about your identity. Each person in your group is indicated by a letter. The other five persons in your group will be indicated by the letters **A, B, C, D** and **E**. You will receive the name **Me**. The same letter always refers to the same person. For your convenience we will call these participants **your neighbors**.

This part of the experiment consists of 30 rounds. In each round you can earn points. Your total earnings in this part of the experiment is the sum of your earnings in each of the 30 rounds. In each round, you - and each other person in your group - has to make one decision. You have to make a **decision** called **color**. This decision is explained in detail below.

Note: during all 30 rounds the other participants in your group will stay the same persons.

Decision (in one single round)

Decision: color Each person in your group has to choose between two colors: **blue** and **green**. As explained above, you interact with each of your neighbors. In these interactions you can earn points. The color chosen by you and the color chosen by your neighbors determine how much you and your neighbors earn in these interactions.

Your earnings and the earnings of one of your neighbors are determined as follows:

your color	color of your neighbor	your earnings	earnings of your neighbor
**blue**	**blue**	95 points	95 points
**blue**	**green**	5 points	90 points
**green**	**blue**	90 points	5 points
**green**	**green**	75 points	75 points

In each round you will interact with **each** participant in your group. Therefore, your total earnings in a round is the sum of all points you earn in each of the interactions. **Note:** you can not choose different colors for different participants. You can, however, choose different colors in different rounds.

You now receive information about the computer screen that you will see during this part of the experiment. You received a print-out of the computer screen (Example screen 1) from us. Take this print-out in front of you. The screen consists of five windows: **round, decision, decision: color, information** and **short explanation**.

- **Round:** This window holds information about past round(s). At the beginning of a new round you will automatically receive information in this window about decisions in the previous round, (In the example, this is round 3; see upper left corner). In the window there are 6 little squares, named **Me, A, B, C, D** and **E**. **Me** always refers to you. The letters refer to the other five persons in your group. This window also shows the interaction structure. This structure remains fixed throughout all 30 rounds.
  - The squares are either **blue** or **green**. This gives you information about the color choices of the persons in your group. (In the example: persons **A**, **B** and **D** have chosen the color **green**, while the persons **Me**, **C** and **E** have chosen the color **blue**)
  - At the bottom of this window you find two buttons called **previous** and **next**. You can use these buttons to look at the decisions in all previous rounds. Between the buttons you can find your **earnings** (in points) in the corresponding round.
- **Decision:** This window is of no importance in the experiment.
- **Decision: color:** This window is located in the lower right corner. In this window you choose between the two colors **blue** and **green**. You make your decision by clicking in the empty button to the left of **Blue** or **Green**.

  When you are satisfied with your decision you have to confirm these decisions by clicking on the button **'Confirm'**.

- **Information:** In this window you find information on the current round and on your **total earnings** up until this round.
- **Short explanation:** If this window turns yellow you have to make your decision. After you made and confirmed your decision this window will turn grey. You then have to wait until all participants are ready. Only then a new round will begin.

Information: After each round, you will receive information about the choices of all persons in your group. All other persons in your group will also receive information about all your decisions.

This is the end of the instructions of part II. You will now have to answer a few questions to make sure that you understood the instructions properly. Thereafter, there will be one practice round in which you cannot yet earn any money. Your decisions in this practice round will not be revealed to other participants. Only after the practice round is over part II of the experiment will begin.

If you have any questions please raise your hand. If you have no questions any more, click on **'Next'**.

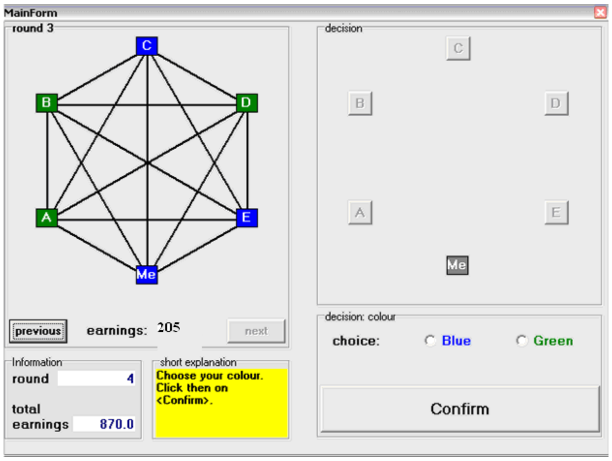

Figure G.2. Example screen 1

Instructions part II (treatment Free)

In this part of the experiment every participant is in a **group of six** with five other participants. Before part II begins, these groups of six will be formed arbitrarily with the help of the computer. The group you are in will not change during this part of the experiment. You will not get information about the identity of the persons in your group, neither during the experiment, nor after the experiment. Other participants will also not receive any information about your identity. Each person in your group is indicated by a letter. The other five persons in your group will be indicated by the letters **A, B, C, D** and **E**. You will receive the name **Me**. The same letter always refers to the same person.

This part of the experiment consists of 30 rounds. In each round you can earn points. Your total earnings in this part of the experiment is the sum of your earnings in each of the 30 rounds. In each round, you

- and each other person in your group - has to make two decisions. You have to make a **decision** called **connections** and a **decision** called **color**. These decisions are explained in detail below.

Note: during all 30 rounds the other participants in your group will stay the same persons.

Decision (in one single round)

Decision: connections You have to decide with whom you want to make a connection. You can make a connection with any other person in your group and you can make as many connections as you want. These choices - together with the choices of the other persons in your group - determine with whom you **interact** (in the respective round) as explained below:

- You will interact with a person with whom you made a connection *and* who made a connection to you. So **mutual consent** is needed for interaction.
- You will not interact if either only you or only the other person made a connection.
- You will not interact with a person if neither of you made a connection with each other.

For your convenience we will call those persons in your group with whom you **interact**, **your neighbors**. Your neighbors are therefore those persons with whom you made a connection and who at the same time also made a connection with you.

Decision: color Each person in your group has to choose between two colors: **blue** and **green**. As explained above, you **interact with all your neighbors**. In these interactions you can earn points. The color chosen by you and the color chosen by your neighbors determine how much you and your neighbors earn in these interactions.

Your earnings and the earnings of one of your neighbors are determined as follows:

your color	color of your neighbor	your earnings	earnings of your neighbor
**blue**	**blue**	95 points	95 points
**blue**	**green**	5 points	90 points
**green**	**blue**	90 points	5 points
**green**	**green**	75 points	75 points

In each round you will **interact with all your neighbors**. Therefore, your total earnings in a round from the interactions with your neighbors is the sum of all points you earn in each of the interactions. For each person in your group with whom you do not interact (i.e., all persons who are not your neighbors) you earn 0 points. For example, if you have no neighbors in a round, then you earn 0 points in this round. **Note:** you can not choose different colors for different participants. You can, however, choose different colors in different rounds.

You now receive information about the computer screen that you will see during this part of the experiment. You received a print-out of the computer screen (Example screen 1) from us. Take this print-out in front of you. The screen consists of five windows: **round, decision: connection, decision: color, information** and **short explanation**.

- **Round:** This window holds information about past round(s). At the beginning of a new round you will automatically receive information in this window about decisions in the previous round. (In the example, this is round 6; see upper left corner.) In the window there are 6 little squares, named **Me, A, B, C, D** and **E**. **Me** always refers to you. The letters refer to the other five persons in your group.
  - A thick full line between two persons (letters or 'Me' indicates that they both made a connection with each other, that is they had an interaction with each other. See, e.g., the line between **Me** and **C** on the example screen).
  - A thin line between two persons indicates that only one of them made a connection: such a line is full on the side of the person that made the connection, and broken on the side of the person that did not make the connection. (See, e.g., the line between **Me** and **B** on the example screen: **Me** made a connection with **B**, but **B** did not make a connection with **Me**.)
  - No line between two persons indicates that neither of them made a connection.
  - The squares are either **blue** or **green**. This gives you information about the color choices of the persons in your group. (In the example: persons **A**, **B** and **D** have chosen the color **green**, while the persons **Me**, **C** and **E** have chosen the color **blue**.)
  - At the bottom of this window you find two buttons called **previous** and **next**. You can use these buttons to look at the decisions in all previous rounds. Between the buttons you can find your **earnings** (in points) in the corresponding round.
- **Decision: connections:** In this window you can choose with whom you want to make a connection. Again, there are five squares named **A, B, C, D** and **E** and the square **Me**. You can make a connection with another person in your group by clicking on the corresponding square. A non-broken grey line will appear to indicate that you made a connection. To remove a connection you made, click on the corresponding square again. (On the example screen: **Me** made a connection with persons **A, B, C** and **D**.)
  Note: At the beginning of each round you will always see the connections that you made in the previous round. If you want, you can remove these connections in the way described above.
- **Decision: color:** This window is located precisely underneath the window, just described. In this window you choose between the two colors **blue** and **green**. You make your decision by clicking in the empty button to the left of **Blue** or **Green**.

  When you are satisfied with all your decisions (that is, with **both**: the **connections** you made and the chosen **color**), you have to confirm these decisions by clicking on the button **'Confirm'**.
- **Information:** In this window you find information on the current round and on your **total earnings** up until this round.
- **Short explanation:** If this window turns yellow you have to make your decision. After you made and confirmed your decision this window will turn grey. You then have to wait until all participants are ready. Only then a new round will begin.

Information: After each round, you will receive information about the choices of all persons in your group. All other persons in your group will also receive information about all your decisions.

This is the end of the instructions of part II. You will now have to answer a few questions to make sure that you understood the instructions properly. Thereafter, there will be one practice round in which you cannot yet earn any money. Your decisions in this practice round will not be revealed to other participants. Only after the practice round is over part II of the experiment will begin.

If you have any questions please raise your hand. If you have no questions any more, click on **'Next'**.

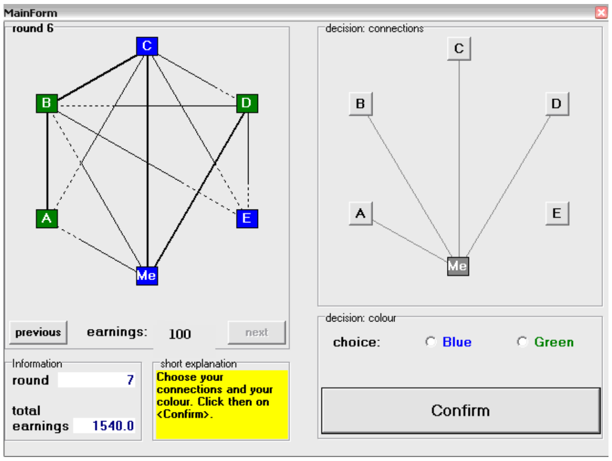

Figure G.3. Example screen 1

Instructions part III

This part of the experiment is exactly the same as the previous part except for the fact that **new groups** of six will arbitrarily be formed with the help of the computer. That is, the composition of the group you are in differs from the composition of the group you were in, in the previous part. During this part of the experiment, the group you are in will not change. This part of the experiment consists of thirty rounds.

Instructions part IV

In this part of the experiment you will be asked to make ten decisions on lotteries.

You received a print-out of the computer screen for part IV from us. Please take this print-out in front of you. We shall explain your choice options with the help of the print-out.

The print-out shows ten decisions. Each decision is a paired choice between "Option A" and "Option B." You will make ten choices, but only one of them will be used in the end to determine your earnings. These choices will affect your earnings, in the following way. After you have made all of your choices, that is after selecting Option A or Option B in each decision row, you will have to click on the button "confirm." Thereafter, a button "start computer" will appear. This button starts the procedure in which the computer randomly selects one of the ten decisions that will be relevant for your earnings. Thereafter,

you will see a new screen where the actual lottery, that is the choice option associated with the selected decision row, will take place. When you press the button "start counting" the computer will randomly pick a number between 1 and 10 which will determine your payoff for the option you chose, Option A or Option B, for the selected decision.

Note: Even though you will make ten decisions, only one of these will actually affect your earnings, but you will not know in advance which decision will be used. Obviously, each decision has an equal chance of being used in the end.

Now, please look at Decision 1 at the top of the example screen. Option A pays 2000 points if the randomly chosen number by the computer is 1, and it pays 1600 points if the randomly chosen number by the computer is 2-10. Option B yields 3850 points if the randomly chosen number by the computer is 1, and it pays 100 points if the randomly chosen number by the computer is 2-10. In Decision 2, Option A pays 2000 points if the randomly chosen number by the computer is 1-2, and it pays 1600 points if the randomly chosen number by the computer is 3-10. Option B yields 3850 points if the randomly chosen number by the computer is 1-2, and it pays 100 points if the randomly chosen number by the computer is 3-10. The payments are determined in a similar manner for the other rows.

If you have any questions please raise your hand. If you have no questions any more, press **'ready'**.

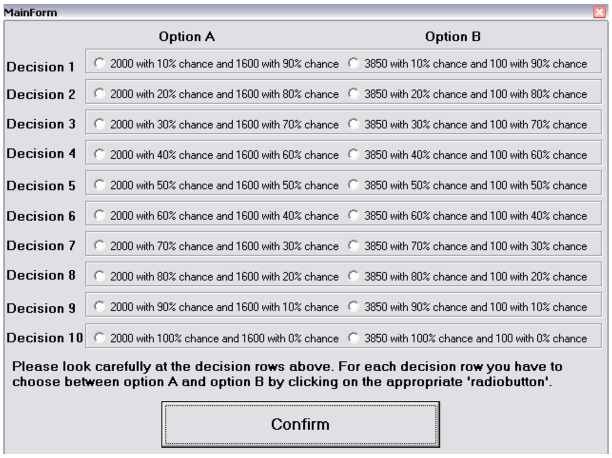

Figure G.4. Example screen: Ten-paired lottery-choice decisions

References

[1] Charles A. Holt and Susan K. Laury. Risk aversion and incentive effects. *American Economic Review*, 92(5):1644–1655, 2002.

[2] Joep Sonnemans, Frans van Dijk, and Frans van Winden. On the dynamics of social ties structures in groups. *Journal of Economic Psychology*, 27(2):187–204, 2006.

[3] Paul A.M. van Lange. The pursuit of joint outcomes and equality in outcomes: An integrative model of social value orientation. *Journal of Personality and Social Psychology*, 77(2):337–349, 1999.
